# A Survey of Lattice-Based Physical-Layer Security for Wireless Systems with *p*-Modular Lattice Constructions†

**DOI:** 10.3390/e28020235

**Published:** 2026-02-18

**Authors:** Hassan Khodaiemehr, Khadijeh Bagheri, Amin Mohajer, Chen Feng, Daniel Panario, Victor C. M. Leung

**Affiliations:** 1 School of Engineering, Faculty of Applied Science, University of British Columbia (UBC), Okanagan Campus, Kelowna, BC V1V 1V7, Canada; hassan.khodaiemehr@ubc.ca (H.K.); khadijeh.bagheri@ubc.ca (K.B.); amin.mohajer@ubc.ca (A.M.); 2 School of Mathematics and Statistics, Carleton University, Ottawa, ON K1S 5B6, Canada; daniel@math.carleton.ca; 3 Department of Electrical and Computer Engineering, University of British Columbia (UBC), Vancouver, BC V6T 1Z4, Canada; vleung@ieee.org

**Keywords:** physical-layer security, Gaussian wiretap channel, wireless communications, lattice codes, modular lattices, cyclotomic fields, secrecy gain, flatness factor, indefinite theta series

## Abstract

Physical-layer security (PLS) provides an information-theoretic framework for securing wireless communications by exploiting channel and signal-structure asymmetries, thereby avoiding reliance on computational hardness assumptions. Within this setting, lattice codes and their algebraic constructions play a central role in achieving secrecy over Gaussian and fading wiretap channels. This article offers a comprehensive survey of lattice-based wiretap coding, covering foundational concepts in algebraic number theory, Construction A over number fields, and the structure of modular and unimodular lattice families. We review key secrecy metrics, including secrecy gain, flatness factor, and equivocation, and consolidate classical and recent results to provide a unified perspective that links wireless-channel models with their underlying algebraic lattice structures. In addition, we review a newly proposed family of *p*-modular lattices in Khodaiemehr, H., 2018 constructed from cyclotomic fields $Q(\zeta_{p})$ for primes p≡1(mod4) via a generalized Construction A framework. We characterize their algebraic and geometric properties and establish a non-existence theorem showing that such constructions cannot be extended to prime-power cyclotomic fields $Q(\zeta_{p^{n}})$ with n>1. Finally, motivated by the fact that these *p*-modular lattices naturally yield mixed-signature structures for which classical theta series diverge, we integrate recent advances on indefinite theta series and modular completions. Drawing on Vignéras’ differential framework and generalized error functions, we outline how modularly completed indefinite theta series provide a principled analytic foundation for defining secrecy-relevant quantities in the indefinite setting. Overall, this work serves both as a survey of algebraic lattice techniques for PLS and as a source of new design insights for secure wireless communication systems.

## 1. Introduction

The relentless evolution of wireless communication systems, characterized by increasing demands for spectral efficiency, data rates, and robust connectivity, necessitates a parallel advancement in security paradigms. Traditional cryptographic methods, while foundational for secure communication, often confront inherent limitations in dynamic wireless environments. These include significant computational overhead, complex key management protocols, and susceptibility to sophisticated attacks such as the man-in-the-middle exploit [1,2]. Consequently, there has been a surge in interest towards physical-layer security, an approach that leverages the intrinsic physical properties of the wireless channel itself to establish security guarantees. PLS operates at the physical layer, providing a complementary layer of defense rooted in information theory, distinct from algorithmic cryptography [3,4].

Historically, algebraic techniques have proven to be unexpectedly powerful tools in various facets of communication system design. Initially, elementary number theory laid the groundwork for error-correcting codes, while finite fields became the mathematical engine for designing powerful binary and non-binary codes. As digital receiver processing capabilities advanced, attention shifted to signal-space codes, where the theory of Euclidean lattices emerged as a cornerstone for constructing dense signal constellations. These lattices facilitated efficient transmission over additive white Gaussian noise (AWGN) channels, with their efficiency often quantified by the volume-to-noise ratio (VNR). The pursuit of capacity-achieving and sphere-bound-achieving lattices spurred significant research efforts [5,6,7]. This led to the development of various practically decodable lattice families, characterized by sparse parity-check matrices that enabled high-dimensional decoding [8,9,10,11,12,13]. Other notable constructions include low-density lattice codes (LDLC) [14], integer low-density lattices based on Construction A (LDA) [15], and polar lattices [16]. Beyond core communication, algebraic methods, such as Gröbner bases, have also found applications in generalized lattice forms and algebraic decoding algorithms [17,18].

The application of algebraic structures extended further with the advent of wireless communication over fading channels. New code design criteria, focused on improving performance in bandwidth-limited scenarios, emerged. Algebraic number theory proved instrumental in this context, facilitating the design of robust coding schemes. These often manifested as multi-dimensional lattice signal constellations, achieving high coding gain through *modulation diversity* (or signal space diversity) [19]. This involved designing intrinsic high-diversity algebraic lattices via canonical embeddings of number fields or applying specific rotations to quadrature amplitude modulation (QAM) constellations. Furthermore, the theoretical exploration of multi-antenna wireless communication in the late 1990s revealed the critical role of division algebras in space–time coding. Once performance criteria for space–time codes were cast as matrix design problems, division algebras, including number fields and cyclic algebras, naturally emerged as optimal solutions, satisfying various performance metrics [20,21,22,23,24,25,26,27]. Other advanced algebraic structures, such as Clifford algebras [28] and crossed product algebras [29], have also been investigated.

Building upon this rich foundation, the focus has more recently shifted to leveraging these advanced algebraic techniques for physical-layer security. The open nature of wireless mediums, the decentralized nature of many networks, and the dynamic topology of mobile systems render traditional cryptographic approaches challenging for secure communication. This context underscores the significance of the information-theoretic approach, initiated by Wyner [3] and Csiszár and Körner [4], which conceptualizes the *wiretap channel* as a scenario where a legitimate receiver (Bob) has a superior channel to an eavesdropper (Eve). This framework demonstrates that reliable and confidential communication is achievable by exploiting physical channel differences without reliance on cryptographic keys. Extensive research has since characterized secrecy capacity across various wiretap channel models, including Gaussian point-to-point and relay networks [1,30].

Within the domain of Gaussian wiretap channels, where secrecy capacity is well-established [31], lattice codes have emerged as a powerful paradigm. Early designs focused on binary inputs [32,33], but a more sophisticated approach introduced by Belfiore and Oggier [34] proposed lattice codes optimized via novel invariants like *secrecy gain* and *flatness factor*. These metrics precisely quantify the eavesdropper’s confusion and the code’s ability to obscure information. A vanishing flatness factor (or equivalently, an infinitely large secrecy gain) is directly linked to robust semantic security [35]. The investigation of these metrics for *ℓ*-modular lattices, particularly unimodular lattices, has been a key research thrust [36,37]. The Belfiore–Solé conjecture on weak secrecy gain sparked rigorous methods for its verification [38,39], with studies extending to the asymptotic behavior, achievable bounds, and specific classifications of unimodular and modular lattices in various dimensions [30,37,40,41,42,43,44,45].

A versatile methodology for constructing lattices with desired properties is Construction A [46]. Its efficacy was amplified through generalization to number fields, initially proposed by Ebeling using cyclotomic fields [47], and subsequently extended to complex multiplication (CM) fields and totally real number fields [48,49]. This generalization provided a framework for lattice coset encoding in wireless channels, including wiretap scenarios [19,49,50], and facilitated the design of lattices with inherent properties like modularity and large shortest vectors, crucial for coding applications and physical network coding [51]. Extensive secrecy gain analysis for *ℓ*-modular lattices from quadratic extensions further underscores the utility of this approach [52,53].

This paper investigates the advanced application of algebraic number theory for constructing cyclotomic modular lattices, specifically designed for improving physical-layer security in next-generation wireless systems. Our structured investigation begins by detailing the Gaussian wiretap channel model in Section 2. This section provides a technical exposition on lattice properties critical for secure communication, including rigorous definitions of secrecy gain and flatness factor. Section 3 introduces the foundational mathematical prerequisites in algebraic number theory. We explore cyclotomic number fields, a class of monogenic fields with predictable algebraic structures, and describe their role in cryptographic and information security contexts. We subsequently examine the Construction A framework, focusing on its application for lattices over general number fields in Section 4. In Section 5, we address the application of cyclotomic number fields in the construction of modular lattices. A central contribution of this work lies in the rigorous construction and characterization of families of *p*-modular lattices derived from cyclotomic number fields. In Section 6, we present explicit construction methodologies utilizing the generalized Construction A framework and review recent theoretical results that precisely determine the conditions under which such modular lattices can exist. These results are not merely of theoretical interest; they yield concrete engineering design rules that provide a clear roadmap for system architects. For example, based on these results, one can demonstrate that constructing modular lattices via Construction A over cyclotomic fields of prime-power order $p^{n}$ is generally infeasible when n>1, while simultaneously proving feasibility and strong structural guarantees for fields of prime order *p* with p≡1(mod4). These insights illuminate both promising future directions and fundamental limitations in algebraic lattice construction for secure communications. Interestingly, these *p*-modular constructions naturally give rise to lattices of mixed signature, for which classical secrecy metrics such as secrecy gain and flatness factor, which are well understood in the positive-definite setting, are no longer directly applicable, as the associated theta series diverges. This challenge motivates a deeper examination of recent advances in the theory of indefinite theta series and their modular completions, particularly the framework introduced by Vignéras and extended through generalized error functions [54]. These developments provide a principled analytic foundation for studying secrecy properties of indefinite lattices and open new future pathways for defining secrecy-relevant quantities beyond the positive-definite regime. To bridge these mathematical advances with physical-layer security, this paper outlines the role of modular completions, Vignéras’ operator, and generalized error functions in enabling meaningful analysis of mixed-signature lattice codes [55,56].

Finally, we transition from theoretical exposition to practical relevance by linking these advanced algebraic constructions to the cutting-edge landscape of modern wireless systems in Section 7. We explore how the unique algebraic structure and inherent properties of our cyclotomic modular lattices—their specific modularity, predictable behavior, and intrinsic potential for optimized secrecy—render them highly suitable for deployment in next-generation security solutions. This includes a detailed discussion of their integration into sophisticated multiple-input multiple-output (MIMO) systems, their synergistic potential with reconfigurable intelligent surfaces (RIS) for dynamic channel shaping, and their role within machine learning (ML)-assisted communication paradigms. This section identifies concrete design benefits, illustrates how our theoretical properties translate into tangible performance gains, and illuminates crucial future research directions for leveraging these robust algebraic constructions in the context of wireless systems. Section 8 offers our concluding remarks and summarizes the broader implications of this work.

## 2. Wireless Wiretap Channels and Secrecy Metrics

This section establishes the fundamental models and performance metrics essential for understanding PLS in wireless communication systems. We begin by reviewing the canonical wireless wiretap channel models, from basic AWGN scenarios to more complex fading environments. Subsequently, we delve into the information-theoretic foundations of secrecy, defining key concepts such as secrecy capacity and equivocation. The section then introduces entropy-based metrics, specifically secrecy gain and flatness factor, which are crucial for quantifying the security performance of lattice codes. Finally, we discuss lattice coset coding as a practical technique for simultaneously achieving reliability and secrecy. This framework will serve as the unifying basis for the subsequent algebraic constructions and analyses presented in this paper.

### 2.1. Wireless Wiretap Models: AWGN and Quasi-Static Fading

**Figure 1:**
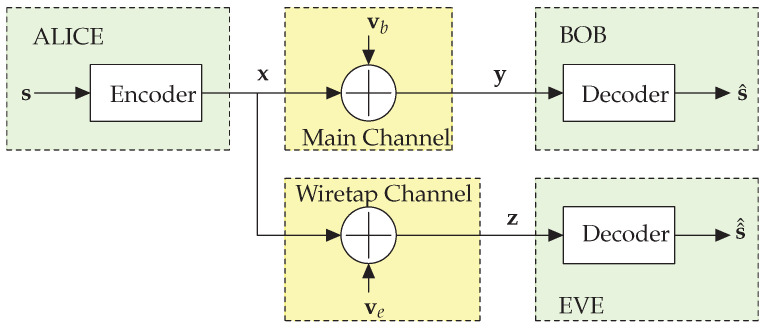
Gaussian wiretap channel setup.

Effective PLS design requires a precise understanding of the underlying wireless channel characteristics. The simplest yet fundamental model is the *Gaussian wiretap channel*, where a legitimate receiver (Bob) and an eavesdropper (Eve) experience independent additive white Gaussian noise. In this scenario, Alice transmits a signal *x*, which is received by Bob as *y* and by Eve as *z*. This model is represented as

(1) $$\begin{array}{c} y=x+v_{b},\\ z=x+v_{e}, \end{array}$$

where *x* is the transmitted signal, $v_{b}$ and $v_{e}$ denote the Gaussian noise at Bob and Eve’s side, respectively, both with zero mean, and respective variances $\sigma_{b}^{2}$ and $\sigma_{e}^{2}$ (see Figure 1).

A more realistic scenario for wireless communications involves *fading channels*, where the channel gain fluctuates over time and space. We primarily consider *quasi-static fading* models, where the channel coefficients remain constant over a coherence interval but vary across different intervals. This distinction is critical as fading can be either exploited or mitigated for secrecy purposes. For the quasi-static fading channel, Alice transmits a signal *x*, which is received by Bob and Eve via channel gains $h_{B}$ and $h_{E}$, respectively, further corrupted by AWGN. The fading channel is modeled as

(2) $$\begin{array}{c} y=h_{B}x+v_{b},\\ z=h_{E}x+v_{e}, \end{array}$$

where $h_{B}$ and $h_{E}$ are the constant (within a coherence interval) channel gains for Bob and Eve, respectively, and $v_{b}$, $v_{e}$ are zero-mean AWGN with variances $\sigma_{b}^{2}$ and $\sigma_{e}^{2}$.

In both AWGN and fading contexts, the fundamental assumption for PLS is usually that Bob possesses a superior channel quality compared to Eve. Specifically, we assume that Alice knows Bob’s channel, that is $\sigma_{b}$; however, Eve’s channel, $\sigma_{e}$, can be known or unknown [37] to Alice. The goal is to achieve reliable communication between Alice and Bob, but also confidentiality despite the presence of Eve. As usual in information security, we assume that Bob has a good SNR (signal to noise ratio) but Eve has a poor SNR, in particular with respect to Bob; that is $\sigma_{e}^{2}\ll \sigma_{b}^{2}$.

Alice’s encoder maps *l* bits $s_{1},\ldots ,s_{l}$ from $S=\left\{0,1\right\}$ to a codeword $x=\left(x_{1},\ldots ,x_{n}\right)\in R^{n}$. Over a transmission of *n* symbols, we get

(3) $$\begin{array}{c} y=x+v_{b},\\ z=x+v_{e}. \end{array}$$

### 2.2. Information-Theoretic Secrecy: Capacity, Equivocation, Mutual Information

The ultimate goal of PLS is to achieve information-theoretic secrecy, ensuring that the legitimate recipient can reliably decode the message while the eavesdropper gains negligible information. This is formally quantified through several key metrics. The information theoretic approach to achieving secure communication was initiated by Wyner [3] and Csiszár and Körner [4], introducing the *wiretap channel* model. *Secrecy capacity* represents the maximum rate at which information can be transmitted reliably and confidentially. For a wiretap channel, it is defined as the difference between the legitimate channel capacity and the eavesdropper’s channel capacity. Extensive research has since characterized secrecy capacity across various wiretap channel models, including Gaussian point-to-point and relay networks [1,40]. The secrecy capacity of Gaussian wiretap channels was established in [31].

To measure how efficient wiretap coding is in providing confidentiality, the information leaked to the eavesdropper is measured in terms of *equivocation* (conditional entropy), that is $H(S^{l}|Z^{n})$, where *S* and *Z* denote random variables corresponding, respectively, to Alice’s data and the message received by Eve. The best possible secrecy is achieved when $H\left(S^{l}|Z^{n}\right)=H\left(S^{l}\right)$, or equivalently when $I\left(S^{l};Z^{n}\right)=H\left(S^{l}\right)-H\left(S^{l}|Z^{n}\right)=0$. It was shown in [35], Theorem 5, for the Gaussian wiretap channel that

(4) $$I\left(S^{l};Z^{n}\right)\leq \epsilon_{n}\left(nR-\log\epsilon_{n}\right),$$

where

(5) $$\epsilon_{n}=\frac{\mathrm{vol}\left(\Lambda_{e}\right)\Theta_{\Lambda_{e}}\left(1/2\pi \sigma_{e}^{2}\right)}{{\left(\sqrt{2\pi \sigma_{e}^{2}}\right)}^{n}}-1;$$

in which vol and Θ are defined in (8) and (14), respectively. For more details about $\epsilon_{n}$ and its properties, the reader may consult [35].

### 2.3. Entropy-Based Metrics: Secrecy Gain and Flatness Factor

While secrecy capacity and equivocation provide fundamental bounds, specific metrics are needed for the practical design and analysis of lattice codes. For lattice-based systems, two critical entropy-based metrics are used to quantify the confusion at the eavesdropper. A different approach was adopted in [34], where lattice codes were proposed using as design criterion a new lattice invariant called *secrecy gain* defined as the maximum of its secrecy function; it was shown that it characterizes the confusion at the eavesdropper. In [35], the result concerning semantic security was generalized to continuous channels, and a new lattice design criterion, designated as the *flatness factor*, was proposed. This study established that a vanishing flatness factor (or equivalently an infinitely large secrecy gain) implies semantic security. This suggests the study of the secrecy gain of lattices as a way to understand how to design good Gaussian lattice wiretap codes.

These metrics are defined formally based on the lattice’s theta series. Before formally defining the theta series, let us establish the fundamental definitions of a lattice and its associated geometric properties.

**Definition** **1.** *A lattice Λ is a discrete additive subgroup of the m-dimensional real space $R^{m}$. It can be generated by a basis $B=\left\{b_{1},\ldots ,b_{n}\right\}\subseteq R^{m}$, where n≤m. Any vector x∈Λ can be represented as an integer linear combination of the basis vectors. The n×m matrix M with $b_{1},\ldots ,b_{n}$ as rows is a generator matrix for the lattice. The rank of the lattice is n and its dimension is m. If n=m, the lattice is a full-rank lattice. A lattice Λ can be formally described as*

(6) $$\Lambda =\left\{x=\mathrm{uM}\;|\;u\in Z^{n}\right\}.$$

For any lattice point p of a lattice $\Lambda \subset R^{m}$, its *Voronoi cell* is defined by

(7) $$V_{\Lambda }\left(p\right)=\left\{x\in R^{m},\;d\left(x,p\right)\leq d\left(x,q\right)\;\;\mathrm{for}\;\;\mathrm{all}\;\;q\in \Lambda \right\}.$$

All Voronoi cells are congruent; thus, $V_{\Lambda }\left(p\right)=V_{\Lambda }\left(0\right)≜V\left(\Lambda \right)$. The *Gram matrix* of a lattice is given by $G=MM^{t}$.

**Definition** **2.** *The volume of a lattice Λ, denoted vol(Λ), is the volume of its fundamental Voronoi cell. It is mathematically defined as the square root of the determinant of its Gram matrix:*

(8) $$\mathrm{vol}\left(\Lambda \right)=\mathrm{vol}\left(V\left(\Lambda \right)\right)=\sqrt{\det(G)}.$$

A lattice Λ in $R^{m}$ is an *integral lattice* if its Gram matrix has coefficients in Z. This means that the inner product $\langle x,y\rangle \in Z$ for all x,y∈Λ. The set of all vectors in $R^{m}$ whose inner product with all vectors of Λ is in Z forms the *dual lattice* of Λ, denoted by $\Lambda^{*}$.

The minimum non-zero squared Euclidean norm of a lattice, denoted $d_{\min}^{2}\left(\Lambda \right)$, and its associated *Hermite parameter*, $\gamma_{H}\left(\Lambda \right)$, are critical for coding performance:

(9) $$\gamma_{H}\left(\Lambda \right)={\left(\frac{d_{\min}\left(\Lambda \right)}{\sqrt[{n}]{\mathrm{vol}(\Lambda )}}\right)}^{2}=\frac{d_{\min}^{2}\left(\Lambda \right)}{\sqrt[{n}]{\det(G)}}.$$

The Hermite parameter quantifies the density of the lattice packing. The number of vectors in Λ with squared norm equal to $d_{\min}^{2}\left(\Lambda \right)$ is the *kissing number* τ(Λ). These definitions are fundamental to understanding the subsequent formulation of the theta series, which provides a comprehensive spectral representation of the lattice’s structure.

Building on the preceding definitions, the *secrecy function* $\Xi_{\Lambda }\left(\tau \right)$ of an *n*-dimensional lattice Λ is given by

(10) $$\Xi_{\Lambda }\left(\tau \right)=\frac{\Theta_{\sqrt[{n}]{\mathrm{vol}(\Lambda )}Z^{n}}\left(\tau \right)}{\Theta_{\Lambda }\left(\tau \right)},\;\tau =yi,\;\;y>0.$$

The *strong secrecy gain* $\chi_{\Lambda ,\mathrm{strong}}$ of an *n*-dimensional lattice Λ is defined by

(11) $$\chi_{\Lambda ,\mathrm{strong}}=\underset{y>0}{\sup}\Xi_{\Lambda }\left(yi\right).$$

Since the above maximum value is not easy to calculate for a general lattice, a weaker definition of secrecy gain has been introduced [37]. A multiplicative symmetry point is a point $y_{0}$ such that $\Xi_{\Lambda }\left(y_{0}\cdot y\right)=\Xi_{\Lambda }\left(y_{0}/y\right)$ for all y>0. The *weak secrecy gain* $\chi_{\Lambda }$ of a lattice Λ is then given by

(12) $$\chi_{\Lambda }=\Xi_{\Lambda }\left(y_{0}\right)=\frac{\Theta_{\sqrt[{n}]{\mathrm{vol}(\Lambda )}Z^{n}}\left(y_{0}i\right)}{\Theta_{\Lambda }\left(y_{0}i\right)}.$$

For an *n*-dimensional *d*-modular lattice, the weak secrecy gain $\chi_{\Lambda }$ is given by [37]:

(13) $$\chi_{\Lambda }=\frac{\Theta_{\sqrt[{4}]{d}Z^{n}}\left(\tau \right)}{\Theta_{\Lambda }\left(\tau \right)},\;\tau =\frac{i}{\sqrt{d}},$$

noting that the volume of a *d*-modular lattice is $\mathrm{vol}\left(\Lambda \right)=d^{\frac{n}{4}}$. The secrecy gain characterizes the amount of confusion that a wiretap lattice code brings [37]. The weak secrecy gain $\chi_{\Lambda }$ is conjectured to be the secrecy gain itself. To the best of our knowledge, this conjecture is still open [53], but for large classes of unimodular lattices, it is known to be true [38,43]. The relationship between *d* and $\chi_{\Lambda }$ for *d*-modular lattices has been investigated in [30,40,52,53].

The *theta series* of a lattice Λ, denoted $\Theta_{\Lambda }\left(\tau \right)$, is a fundamental tool for analyzing its properties. For H={a+ib∈C|b>0} (the upper half-plane) and $q=e^{\pi i\tau }$, τ∈H, the theta series is defined by

(14) $$\Theta_{\Lambda }\left(\tau \right)=\sum_{t\in \Lambda }q^{{∥t∥}^{2}},$$

where ${∥t∥}^{2}=\langle t,t\rangle$ is the squared Euclidean norm of a lattice vector, in which $\langle ,\rangle :\Lambda \times \Lambda \to R$ is the bilinear form that Λ is defined based on it. If $\Lambda \subset R^{n}$, we can consider ${∥t∥}^{2}=\sum_{i=1}^{n}t_{i}^{2}$, for $t=(t_{1},\ldots ,t_{n})\in \Lambda$. If we consider Λ to be integral, the theta series of Λ can be written as $\sum_{m\in Z}A_{m}q^{m}$, where $A_{m}=|\{x\in {\Lambda :\;∥x∥}^{2}=m\}|$. For an integral lattice Λ, the coefficient of the second term of $\Theta_{\Lambda }=\sum_{m\in Z}A_{m}q^{m}$, which is $A_{\mu }q^{\mu }$ with

(15) $$\mu =\mu_{\Lambda }=\min\left\{{∥x∥}^{2}\;:\;x\in \Lambda ,\;x\neq 0\right\},$$

is the *kissing number* of Λ, and the power of *q* in the second term gives the *minimum*, or *minimal norm* of Λ. The theta series helps in determining bounds for the minimum [57] as well as classifying lattices [58]. It has also been used recently to define the notion of secrecy gain. Exceptional lattices have theta series that can be expressed as functions of the Jacobi theta functions $\vartheta_{i}\left(q\right)$, $q=e^{\pi i\tau }$, ℑ(τ)>0, i=2,3,4, themselves defined by

(16) $$\begin{array}{ccc} \vartheta_{2}\left(q\right) & = & \sum_{k=-\infty }^{+\infty }q^{{\left(k+\frac{1}{2}\right)}^{2}}, \end{array}$$

(17) $$\begin{array}{ccc} \vartheta_{3}\left(q\right) & = & \sum_{k=-\infty }^{+\infty }q^{k^{2}}, \end{array}$$

(18) $$\begin{array}{ccc} \vartheta_{4}\left(q\right) & = & \sum_{k=-\infty }^{+\infty }{\left(-1\right)}^{k}q^{k^{2}}. \end{array}$$

A few examples of theta series of exceptional lattices are given in Table 1 [37,46].

### 2.4. Lattice Coset Coding: Reliability and Secrecy Analysis

*Lattice coset coding* is a powerful technique for simultaneously achieving high reliability for the legitimate receiver and strong secrecy for the eavesdropper. This method, originally proposed in [3,59], involves mapping an information vector $s_{d}\in {\left\{0,1\right\}}^{k}$ to a coset of a nested lattice structure, rather than a single lattice point. Alice, the transmitter, chooses a point x from the selected coset $\Lambda_{e}+c_{j}\left(s_{d}\right)$ by adding a random vector $r\in \Lambda_{e}$ to the coset representative $c_{j}\left(s_{d}\right)$. The transmitted signal is thus $x=r+c_{j}\left(s_{d}\right)\in \Lambda_{e}+c_{j}\left(s_{d}\right)$.

The underlying principle involves partitioning a dense lattice $\Lambda_{b}$ (intended for Bob) into a union of disjoint cosets of a coarser sublattice $\Lambda_{e}$, such that $\Lambda_{b}={⋃}_{j=1}^{2^{k}}\left(\Lambda_{e}+c_{j}\right)$. The number of such cosets, $2^{k}$, is determined by the ratio of volumes:

(19) $$\left|\frac{\Lambda_{b}}{\Lambda_{e}}\right|=2^{k}=\frac{\mathrm{vol}\left(V(\Lambda_{e})\right)}{\mathrm{vol}\left(V(\Lambda_{b})\right)}.$$

Once the mapping $s_{d}\mapsto \Lambda_{e}+c_{j(s_{d})}$ is done, Alice randomly chooses a point $x\in \Lambda_{e}+c_{j(s_{d})}$ and sends it over the wiretap channel. The coset encoding means that a random vector $r\in \Lambda_{e}$ is chosen and the transmitted lattice point $x\in \Lambda_{b}$ is finally of the form

(20) $$x=r+c_{j(s_{d})}\in \Lambda_{e}+c_{j(s_{d})}.$$

The subscript of the sublattice $\Lambda_{e}$ is chosen since it encodes the random bits that are there to increase Eve’s confusion, and so is the lattice intended for Eve [37]. The total rate *R* is then

(21) $$R=R_{s}+R_{e},$$

where $R_{s}=\frac{2k}{n}$ is the information bits rate intended to Bob, and $R_{e}=\frac{2r}{n}$, with *r* the number of random bits, is the random bit rate, all per (complex) channel use [37].

**Example** **1.** *Based on [34], Example 1, take $\Lambda_{b}=Z^{2}$ and $\Lambda_{e}=2Z^{2}$, for which we have*

$$Z^{2}=2Z^{2}\cup \left(2Z^{2}+\left(0,1\right)\right)\cup \left(2Z^{2}+\left(1,0\right)\right)\cup \left(2Z^{2}+\left(1,1\right)\right).$$

*The lattice $Z^{2}$ is thus partitioned into $2^{k}=4$ cosets, allowing to transmit k=2 bits of information. Alice can label any of the above 4 cosets by their representatives. To transmit the two bits 01, she then randomly picks a point in the coset $2Z^{2}+\left(0,1\right)$, say x=(2,3)=2(1,1)+(0,1) and sends this point over the wiretap channel.*

Using lattice coset encoding, two lattices play a role:

- the lattice $\Lambda_{b}$, that Alice uses to communicate reliably with Bob, and 
- the lattice $\Lambda_{e}$, which is a sublattice of $\Lambda_{b}$, that appears in the process of coset coding for encoding random bits.

In the rest of this section, we present the properties of these two lattices which are related to the design of good wiretap codes. Suppose that the lattice point $x_{k}=r_{k}+c_{k}\in \Lambda_{b}$ has been transmitted over the unconstrained AWGN channel and $y_{k}=x_{k}+v$ has been received, where $v=(v_{1},\ldots ,v_{n})$ is the error term with components independently and identically distributed (i.i.d.) with $N(0,\sigma^{2})$. The probability $P_{c}$ of finding the correct coset is [34]

(22) $$P_{c}=\frac{1}{{\left(\sigma \sqrt{2\pi }\right)}^{n}}\sum_{r\in \Lambda_{e}}\int_{V(\Lambda_{b})+r}e^{-{∥u∥}^{2}/2\sigma^{2}}du.$$

Considering the wiretap channel where Alice transmits lattice codewords from an *n*-dimensional lattice $\Lambda_{b}$, we get that the probabilities $P_{c,b}$ and $P_{c,e}$, which are the correct decision probabilities for Bob and Eve, respectively, can be obtained from (22) by replacing $\sigma =\sigma_{b}$ and $\sigma_{e}$, respectively. Since by assumption Bob has a good SNR, its received vector y is most likely to lie in the Voronoi region around the origin, and thus the terms corresponding to r≠0 in (22) are negligible, which yields [34]

(23) $$P_{c,b}\approx \frac{1}{{\left(\sigma_{b}\sqrt{2\pi }\right)}^{n}}\int_{V(\Lambda_{b})}e^{-{∥u∥}^{2}/2\sigma_{b}^{2}}du.$$

This is now the familiar case of transmitting lattice points over the Gaussian channel, for which it is known that $\Lambda_{b}$ should have a good Hermite parameter to get a good coding gain [46]. We know how to design good codes for Bob’s channel, and have his probability of making a correct decision arbitrarily close to 1 (see, e.g., [7,8,14,60,61]).

On the contrary under a low SNR assumption for Eve, namely $\sigma_{e}$ is large, by using a Taylor expansion, the probability of making a correct decision for Eve is given by [34]

(24) $$P_{c,e}\approx \frac{1}{{\left(\sigma_{e}\sqrt{2\pi }\right)}^{n}}\mathrm{vol}\left(V\left(\Lambda_{b}\right)\right)\sum_{r\in \Lambda_{e}}e^{-{∥u∥}^{2}/2\sigma_{e}^{2}}.$$

In order to minimize the probability $P_{c,e}$ of Eve making a correct decision, while keeping $P_{c,b}$ unchanged, we should find a lattice $\Lambda_{b}$ which is as good as possible for the Gaussian channel, its sublattice $\Lambda_{e}$ minimizes $\sum_{r\in \Lambda_{e}}e^{-{∥u∥}^{2}/2\sigma_{e}^{2}}$ and $\log_{2}\left|\Lambda_{b}/\Lambda_{e}\right|=k$; see [34]. The constraint on the cardinality of cosets (or rate) is equivalent to set the fundamental volume of $\Lambda_{e}$ equal to a constant. Two lattice design criteria have been proposed to characterize the confusion created by $\Lambda_{e}$: the secrecy gain [37], and the flatness factor [35,62]. The secrecy gain originally captures the loss in Eve’s probability of correctly decoding when $\Lambda_{e}$ is used instead of $Z^{n}$ [37]. Both the flatness factor and the secrecy gain involve the theta series of $\Lambda_{e}$ at a particular point, which turns out to give an upper bound on Eve’s knowledge of the secret message (when it is expressed in terms of mutual information) [35]. This concludes our survey of wireless wiretap channels and secrecy metrics, setting the stage for the algebraic construction methods that follow.

## 3. Mathematical Background on Algebraic Number Theory

To systematically engineer lattice codes with tailored properties for wireless communication security, we leverage the powerful tools of algebraic number theory. This section lays out the fundamental definitions and theorems necessary to understand the construction of lattices from number fields. These algebraic structures provide the blueprint for generating lattices with specific characteristics, such as integrality, modularity, and prescribed embedding properties, which are critical for optimizing secrecy metrics.

### 3.1. Number Fields, Dedekind Domains, and Discriminants

We begin with the fundamental definitions concerning number fields, which are finite extensions of the field of rational numbers Q. These fields provide the algebraic setting for constructing advanced lattice codes.

Let *K* and *L* be two fields. If K⊂L, *L* is a field extension of *K* denoted by L/K. The dimension of *L* as a vector space over *K* is the degree of *L* over *K*, denoted by [L:K]. Any finite extension of Q is a *number field*.

An element α∈L is *algebraic over K* if it is a root of a non-zero irreducible monic polynomial p∈K[x]. The polynomial of least degree with this property is known as the *minimal polynomial* of α over *K*. If all elements of *L* are algebraic over *K*, then *L* is an *algebraic extension* of *K*.

**Definition** **3** (Algebraic Integer)**.** *Let K be an algebraic number field of degree n. If α∈K is a root of a monic polynomial with coefficients in Z, then α is an algebraic integer. The set of algebraic integers of K is the ring of integers of K, denoted by $O_{K}$. The ring $O_{K}$ is also called the maximal order of K.*

If *K* is a number field, then K=Q(θ) for an algebraic integer $\theta \in O_{K}$ [63], p. 49. For a number field *K* of degree *n*, the ring of integers $O_{K}$ forms a free Z-module of rank *n*.

**Definition** **4** (Integral Basis)**.** *Let $\left\{\omega_{1},\ldots ,\omega_{n}\right\}$ be a basis of the Z-module $O_{K}$, so that we can uniquely write any element of $O_{K}$ as $\sum_{i=1}^{n}a_{i}\omega_{i}$ with $a_{i}\in Z$ for all i. Then, $\left\{\omega_{1},\ldots ,\omega_{n}\right\}$ is an integral basis for K.*

**Theorem** **1** ([63], p. 41)**.** *Let K=Q(θ) be a number field of degree n over Q. There are exactly n embeddings $\sigma_{1},\ldots ,\sigma_{n}$ of K into C defined by $\sigma_{i}\left(\theta \right)=\theta_{i}$, for i=1,…,n, where $\theta_{i}$’s are the distinct zeros in C of the minimum polynomial of θ over Q.*

For any x∈K, the images $\sigma_{1}\left(x\right),\ldots ,\sigma_{n}\left(x\right)$ are the *conjugates of x*. These conjugates are fundamental for defining two important field-theoretic operations: the norm and the trace.

**Definition** **5** (Norm and Trace)**.** *Let K be a number field of degree n and x∈K. The* norm *of x over Q is defined as $N_{K/Q}\left(x\right)=\prod_{i=1}^{n}\sigma_{i}\left(x\right)$, and the* trace *of x over Q is defined as ${\mathrm{Tr}}_{K/Q}\left(x\right)=\sum_{i=1}^{n}\sigma_{i}\left(x\right)$. For any x∈K, $N_{K/Q}\left(x\right)$ and ${\mathrm{Tr}}_{K/Q}\left(x\right)$ belong to Q. If $x\in O_{K}$, these values are integers, i.e., $N_{K/Q}\left(x\right)\in Z$ and ${\mathrm{Tr}}_{K/Q}\left(x\right)\in Z$.*

**Definition** **6** (Discriminant)**.** *Let $\left\{\omega_{1},\ldots ,\omega_{n}\right\}$ be an integral basis for a number field K. The* discriminant of *K, denoted $d_{K}$, is defined as $d_{K}={\left(\det\left[{\left(\sigma_{j}\left(\omega_{i}\right)\right)}_{i,j=1}^{n}\right]\right)}^{2}$. The discriminant $d_{K}$ of a number field belongs to Z and is independent of the choice of basis.*

**Definition** **7** (Integrally Closed Ring)**.** *A ring A is* integrally closed *in a field L if every element of L which is integral over A lies in A. An integral domain is integrally closed if it is integrally closed in its quotient field.*

**Theorem** **2** ([64], p. 18)**.** *Let D be a ring which is Noetherian, integrally closed, and such that every non-zero prime ideal is maximal. Then, every ideal of D can be uniquely factored into prime ideals.*

A ring satisfying the properties of Theorem 2 is a *Dedekind ring*. Crucially, the ring of algebraic integers $O_{K}$ in any number field *K* is a Dedekind ring.

### 3.2. Cyclotomic Fields: Monogenicity and Galois Structure

*Monogenic number fields* simplify arithmetic within their rings of integers, making them particularly attractive for lattice constructions. Among these, *cyclotomic fields* hold a prominent place due to their well-understood structure and Abelian Galois groups.

**Definition** **8** (Monogenic Number Field)**.** *Let K be a number field of degree n and $O_{K}$ its ring of integers. Then, considering $O_{K}$ as a Z-module, if it has a basis of the form $\left\{1,\alpha ,\ldots ,\alpha^{n-1}\right\}$, for some $\alpha \in O_{K}$, α is a power generator, this basis is a power basis and K is monogenic.*

The problem of identifying monogenic number fields is classical. Quadratic and cyclotomic number fields are known to be monogenic, though this is not a general property for all number fields. For instance, Dedekind [65], p. 64, provided an example of a non-monogenic cubic field. Monogenicity significantly simplifies arithmetic tasks like factoring prime ideals.

Cyclotomic number fields are among the most important monogenic number fields. For any field *K*, an extension of the form K(ζ), where ζ is a root of unity, is a *cyclotomic extension* of *K*. A key algebraic property is that cyclotomic extensions always possess an Abelian Galois group. This makes them essentially the only direct construction method for Abelian extensions over arbitrary base fields [66].

Let $\mu_{n}$ denote the group of *n* different *n*th roots of unity. A primitive *n*th root of unity, denoted $\zeta_{n}$, is an *n*th root of unity that has order *n*.

**Lemma** **1** ([66], Lemma 2.1)**.** *For $\sigma \in \mathrm{Gal}(K\left(\zeta_{n}\right)/K)$ there is an integer $a_{\sigma }$ that is relatively prime to n such that $\sigma \left(\omega \right)=\omega^{a_{\sigma }}$ for all $\omega \in \mu_{n}$.*

Based on Lemma 1, for any field *K*, the mapping $\tau :\mathrm{Gal}\left(K\left(\zeta_{n}\right)/K\right)\to {\left(Z/nZ\right)}^{\times }$, $\sigma \mapsto a_{\sigma }$, is an injective group homomorphism. When K=Q, this embedding is an isomorphism. For n>2, $K=Q(\zeta_{n})$ is a totally imaginary field. It is proved in [67], Theorem 2.6, that $Z[\zeta_{n}]$ is the ring of algebraic integers of $Q(\zeta_{n})$, confirming its monogenicity.

The discriminant of cyclotomic fields can be computed via the following theorem:

**Theorem** **3** ([67], Proposition 2.7)**.** *Let $K=Q(\zeta_{n})$, then the discriminant $d_{K}$ is given by*

(25) $$d_{K}={\left(-1\right)}^{\phi (n)/2}\frac{n^{\phi (n)}}{\prod_{p|n}p^{\phi (n)/(p-1)}}.$$

For the specific case where $n=p^{k}$ (for *p* a prime number), the discriminant of $K=Q(\zeta_{p^{k}})$ is

(26) $$d_{K}=\pm p^{p^{k-1}\left(pk-k-1\right)},$$

where the sign depends on $p^{k}\;\left(\mathrm{mod}\;4\right)$ [67], Proposition 2.1. For $n=2^{r}$ with r>2, $d_{Q(\zeta_{2^{r}})}=2^{2^{r-1}\left(r-1\right)}$ and for r=2, $d_{K}=-4$ [68].

Differences exist between the prime-power case and the general *n* case. For *n* with at least two distinct prime factors, $1-\zeta_{n}$ is a unit of $Z[\zeta_{n}]$ [67], Proposition 2.8. If *n* is prime, however, $1-\zeta_{n}$ is not a unit, and $P=(1-\zeta_{n})$ forms a prime ideal of $O_{K}=Z\left[\zeta_{n}\right]$, with $nO_{K}=P^{n-1}$ signifying total ramification in $Q(\zeta_{n})$ [67], Lemma 1.4.

The *n*th cyclotomic polynomial, $\Phi_{n}\left(X\right)$, is irreducible over Z and defined as

(27) $$\Phi_{n}\left(X\right)=\prod_{(j,n)=1}\left(X-\zeta_{n}^{j}\right).$$

It factors $X^{n}-1=\prod_{d|n}\Phi_{d}\left(X\right)$. The factorization of $\Phi_{n}\left(X\right)$ modulo *p* (for *p* not dividing *n*) yields distinct monic irreducible factors, each with degree equal to the order of p(modn) [66], Theorem 5.4.

**Proposition** **1** ([66], Corollary 5.7)**.** *The reduction ${\bar{\Phi }}_{n}$ is irreducible in $F_{p}\left[X\right]$ if and only if (p,n)=1 and p(modn) is a generator of ${\left(Z/\left(n\right)\right)}^{\times }$.*

### 3.3. Ideals, Embeddings, and Minkowski Space

The theory of ideals within number fields is fundamental for constructing lattices, particularly through Construction A, as it governs the structure of the resulting lattice and its properties.

**Definition** **9** (Signature)**.** *Let $\left\{\sigma_{1},\ldots ,\sigma_{n}\right\}$ be the n embeddings of K into C. Let $r_{1}$ be the number of embeddings whose images lie in R (real embeddings), and $2r_{2}$ be the number of embeddings whose images lie in C∖R (complex embeddings). These values satisfy $r_{1}+2r_{2}=n$. The pair $\left(r_{1},r_{2}\right)$ is called the signature of K.*

- *If $r_{2}=0$, K is a* totally real algebraic number field.
- *If $r_{1}=0$, K is a* totally complex algebraic number field.

The canonical embedding, also known as the Minkowski embedding, is a crucial mapping that translates the algebraic structure of a number field into a concrete geometric representation, laying the foundation for lattice constructions.

We order the embeddings $\sigma_{j}$ such that $\sigma_{j}\left(x\right)\in R$ for $1\leq j\leq r_{1}$, and $\sigma_{j+r_{2}}\left(x\right)=\bar{\sigma_{j}\left(x\right)}$ for $r_{1}+1\leq j\leq r_{1}+r_{2}$.

**Definition** **10** (Canonical Embedding)**.** *The canonical embedding $\sigma :K\to R^{r_{1}}\times C^{r_{2}}$ is the homomorphism defined by*

(28) $$\sigma \left(x\right)=(\sigma_{1}\left(x\right),\ldots ,\sigma_{r_{1}}\left(x\right),\sigma_{r_{1}+1}\left(x\right),\ldots ,\sigma_{r_{1}+r_{2}}\left(x\right)).$$

*By identifying $R^{r_{1}}\times C^{r_{2}}$ with $R^{n}$, the canonical embedding can be rewritten as $\sigma :K\to R^{n}$*

(29) $$\begin{array}{ccc} \sigma (x) & = & (\sigma_{1}\left(x\right),\ldots ,\sigma_{r_{1}}\left(x\right),ℜ\sigma_{r_{1}+1}\left(x\right),ℑ\sigma_{r_{1}+1}\left(x\right),\\ & & \ldots ,ℜ\sigma_{r_{1}+r_{2}}\left(x\right),ℑ\sigma_{r_{1}+r_{2}}\left(x\right)), \end{array}$$

*where $ℜ\sigma_{j}$ denotes the real part of $\sigma_{j}$ and $ℑ\sigma_{j}$ the imaginary part of $\sigma_{j}$, for $j=r_{1}+1,\ldots ,r_{1}+r_{2}$.*

This embedding transforms algebraic integers into lattice points in Euclidean space, allowing us to leverage their geometric properties for coding.

In applications of number fields to lattice construction, understanding the factorization of prime ideals is essential. This process involves selecting an integer $\alpha \in O_{L}$ such that L=K(α), and examining the factorization of its minimal polynomial modulo p.

Let *A* be a Dedekind ring, *K* its quotient field, *L* a finite separable extension of *K*, and *B* the integral closure of *A* in *L*. If p is a prime ideal of *A*, then the ideal pB in *B* can be factored uniquely into prime ideals:

$$pB=P_{1}^{e_{1}}\ldots P_{g}^{e_{g}},$$

where $P_{i}$ are distinct prime ideals of *B* lying above p.

- $e_{i}\geq 1$ is the *ramification index* of $P_{i}$ over p, denoted $e(P_{i}/p)$.
- $f_{i}=\left[B/P_{i}:A/p\right]$ is the *residue class degree* (or inertia degree) of $P_{i}$ over p.

**Theorem** **4** ([64], p. 24)**.** *Let A be a Dedekind ring, K its quotient field, L a finite separable extension of K, and B the integral closure of A in L. Let p be a prime of A. Then, the fundamental identity holds as follows:*

(30) $$\left[L:K\right]=\sum_{P|p}e\left(P/p\right)f\left(P/p\right).$$

For a Galois extension L/K of degree *n*, this simplifies to n=efg, where *g* is the number of primes P of *B* above p, and e=e(P/p) and f=f(P/p) are constant for all P.

- p is *unramified* in *L* if all $e_{i}=1$.
- p is *ramified* if any $e_{i}>1$.
- P is *totally ramified* over p if e(P/p)=[L:K] (implying g=1,f=1).
- p *splits completely* in *L* if $e_{i}=1,f_{i}=1$ for all *i*, so g=[L:K].

These concepts are crucial for understanding the properties of lattices constructed from ideals, particularly in Construction A where the choice of prime ideal p significantly influences the modularity and other characteristics of the resulting lattice.

**Proposition** **2** ([64], p. 27)**.** *Let A be a Dedekind ring with quotient field K and let E be a finite separable extension of K. Let B be the integral closure of A in E and assume that B=A[α] for some element α. Let f be the irreducible polynomial of α over K, p be a prime of A and $\bar{f}$ be the reduction in f modp. Consider*

(31) $$\bar{f}\left(X\right)=\bar{P_{1}}{\left(X\right)}^{e_{1}}\ldots \bar{P_{r}}{\left(X\right)}^{e_{r}},$$

*to be the factorization of $\bar{f}$ into powers of irreducible factors over $\bar{A}=A/p$, with leading coefficients* 1*. Then*

(32) $$pB=P_{1}^{e_{1}}\ldots P_{r}^{e_{r}},$$

*is the factorization of p in B, so that $e_{i}$ is the ramification index of $P_{i}$ over p. We also have*

(33) $$P_{i}=pB+P_{i}\left(\alpha \right)B,$$

*where $P_{i}\in A\left[X\right]$ is a polynomial with leading coefficient* 1 *whose reduction modp is $\bar{P_{i}}$. For each i, $P_{i}$ has residue class degree $\left[B/P_{i}:A/p\right]=d_{i}$, where $d_{i}=deg\left(\bar{P_{i}}\right)$.*

In our cases, A=Z, K=Q, E=Q(α), $B=O_{E}=Z\left[\alpha \right]$ and p=pZ, for a prime number *p*. Proposition 2 shows the importance of monogenic number fields. Indeed, the task of factoring $pO_{K}$, for a prime number *p*, into prime ideals over $O_{K}$ which is a difficult task in general, reduces to factoring the minimal polynomial of α over $F_{q}$, which is significantly easier. Thus, we present other useful results about monogenic number fields.

**Theorem** **5** ([67], Proposition 2.16)**.** *$Z[\zeta_{n}+\zeta_{n}^{-1}]$ is the ring of integers of $Q{\left(\zeta_{n}\right)}^{+}$.*

If $n=p^{r}$, the degree of $K=Q(\zeta_{p^{r}})$ over Q is $p^{r-1}\left(p-1\right)$ and the prime *p* totally ramifies in *K* as $pO_{K}=P^{p^{r-1}\left(p-1\right)}$, where P is a prime principal ideal with generator $1-\zeta_{p^{r}}$ and residue field $O_{K}/P≅F_{p}$. The degree of $K^{+}=Q\left(\zeta_{p^{r}}+\zeta_{p^{r}}^{-1}\right)$ over Q is $\frac{p^{r-1}\left(p-1\right)}{2}$ and it can be proved that the prime *p* also totally ramifies in $K^{+}$ as $pO_{K^{+}}=p^{\frac{p^{r-1}\left(p-1\right)}{2}}$, with $p=P\cap O_{K^{+}}=\left(2-\zeta_{p^{r}}-\zeta_{p^{r}}^{-1}\right)O_{K^{+}}$ [49]. By using the Hasse Theorem that states the conductor-discriminant relation, a formula has been obtained to compute the discriminant of any subfield of $Q(\zeta_{p^{r}})$ where *p* is an odd prime and *r* is a positive integer [69]. Let *p* be an odd prime number, *r* a positive integer, and $L=Q(\zeta_{p^{r}})$. Since *L* is a Galois extension of Q and its Galois group is a cyclic group isomorphic to ${\left(Z/p^{r}Z\right)}^{\times }$, there is a one-to-one correspondence between the subfields of *L* and the divisors of $\left[L:Q\right]=\left(p-1\right)p^{r-1}$. The discriminant of any subfield *K* of *L* can be obtained as a function of *p* and its degree only. Since the degree of *K* is a divisor of $\left(p-1\right)p^{r-1}$, we write $\left[K:Q\right]=up^{j}$, where *u* is a divisor of p−1 and j≤r−1.

**Theorem** **6** ([69], Theorem 4.1)**.** *Let K be a subfield of $Q(\zeta_{p^{r}})$ with $\left[K:Q\right]=up^{j}$, where p∤u. Then, $d_{K}=p^{u\left[\left(j+2\right)p^{j}-\frac{p^{j+1}-1}{p-1}\right]-1}$.*

Another formula for computing the discriminant of any number field $K\subset Q(\zeta_{2^{r}})$, with r≥3, is derived in [68]. In this case the Galois group is ${\left(Z/2^{r}Z\right)}^{\times }=\langle \bar{-1},\bar{5}\rangle =\left\{{\left(\bar{-1}\right)}^{a}{\bar{5}}^{b}|a=1,2\;\;\mathrm{and}\;\;b=1,2,\ldots ,2^{r-2}\right\}$, which is not cyclic.

**Theorem** **7** ([68], Theorem 3.2)**.** *Let K be a subfield of $Q(\zeta_{2^{r}})$ of degree $2^{\left(m-1\right)}$. Then, if $K=Q(\zeta_{2^{m}})$, $d_{K}=2^{2^{m-1}\left(m-1\right)}$ and if $K\neq Q(\zeta_{2^{m}})$, $d_{K}=2^{m2^{m-1}-1}$.*

## 4. Lattices from Number Fields and Construction A

This section details the construction of lattices using algebraic number theory, particularly focusing on the widely used Construction A. This methodology translates algebraic structures into concrete lattice geometries, enabling the design of codes with desired properties for physical-layer security.

### 4.1. Lattices and Modularity

A fundamental understanding of lattices and their modular properties is essential for their application in coding theory and physical-layer security. The definitions of a *lattice*, its *volume*, *Voronoi cell*, *Gram matrix*, *integral lattice*, *dual lattice*, *Hermite parameter*, and *kissing number* have been comprehensively established in Section 2.3, which discusses entropy-based secrecy metrics. Readers are directed there for the detailed mathematical definitions essential for understanding lattice properties. Here, we build upon those foundational concepts to introduce the specialized notions of *unimodular* and *modular lattices*, which are particularly relevant for algebraic constructions in physical-layer security. First, we present the definition of some lattices that arise from algebraic number theory.

**Definition** **11.** *An integral lattice *Γ *is a free Z-module of finite rank together with a positive definite symmetric bilinear form $\langle ,\rangle :\Gamma \times \Gamma \to Z$.*

**Definition** **12.** *The discriminant of a lattice* Γ*, denoted disc(Γ), is the determinant of $MM^{t}$ where M is a generator matrix for* Γ*. The volume $\mathrm{vol}(R^{n}/\Gamma )$ of a lattice *Γ *is defined to be |det(M)|.*

Thus, the discriminant is related to the volume of a lattice by

(34) $$\mathrm{vol}\left(R^{n}/\Gamma \right)=\sqrt{\mathrm{disc}(\Gamma )}.$$

Moreover, when Γ is integral, we have $\mathrm{disc}\left(\Gamma \right)=|\Gamma^{*}/\Gamma |$, where $\Gamma^{*}$ is the dual of the lattice defined by

(35) $$\Gamma^{*}=\left\{y\in R^{m}\;\;|\;\;y\cdot x\in Z\;\;\;\forall \;\;\;x\in \Gamma \right\}.$$

**Definition** **13** (Unimodular and Modular Lattices)**.** When $\Gamma =\Gamma^{*}$, the lattice Γ is unimodular. If (L,b) is integral and $\left(L,b\right)≅\left(L^{*},db\right)$ for some positive integer d, it is d-modular (or modular of level d). An integral lattice (Λ,b) is called even if b(x,x)∈2Z for all x∈Λ and odd otherwise.

The canonical embedding (29) gives a geometrical representation of a number field and makes the connection between algebraic number fields and lattices.

**Theorem** **8** ([63], p. 155)**.** *Let $\left\{\omega_{1},\omega_{2},\ldots ,\omega_{n}\right\}$ be an integral basis for a number field K. The n vectors $v_{i}=\sigma \left(\omega_{i}\right)\in R^{n}$, i=1,…,n are linearly independent, so they define a full rank lattice $\Lambda =\Lambda \left(O_{K}\right)=\sigma \left(O_{K}\right)$.*

**Theorem** **9** ([70])**.** *Let $d_{K}$ be the discriminant of a number field K. The volume of the fundamental parallelotope of $\Lambda (O_{K})$ is given by*

(36) $$\mathrm{vol}\left(\Lambda \left(O_{K}\right)\right)=2^{-r_{2}}\sqrt{|d_{K}|}.$$

### 4.2. Construction A over $O_{K}$

Construction A provides a powerful method to build lattices from linear codes, and its generalization to number fields allows for the creation of lattices with rich algebraic properties suitable for wireless communication.

The generalized Construction A method leverages a number field *K* and a finite field $F_{p^{f}}$. Let *K* be a Galois number field of degree *n* which is either totally real or a CM field. Let $O_{K}$ be the ring of integers of *K* and p be a prime ideal of $O_{K}$ above the prime *p*. We have $O_{K}/p≅F_{p^{f}}$, where *f* is the inertia degree of *p*. The reduction map ρ from $O_{K}^{N}$ to $F_{p^{f}}^{N}$ is defined componentwise as

(37) $$\begin{array}{ccc} \rho :O_{K}^{N} & \to & F_{p^{f}}^{N},\\ \left(x_{1},\ldots ,x_{N}\right) & \mapsto & \left(x_{1}\;\mathrm{mod}\;p,\ldots ,x_{N}\;\mathrm{mod}\;p\right), \end{array}$$

for some positive integer *N*. Let $C\subset F_{p^{f}}^{N}$ be a linear code over $F_{p^{f}}$. Since ρ is a Z-module homomorphism, $\rho^{-1}\left(C\right)$ is a submodule of $O_{K}^{N}$. Given that $O_{K}$ is a free Z-module of rank *n*, $\rho^{-1}\left(C\right)$ is a free Z-module of rank nN. The lattice is then constructed using a symmetric bilinear form $b_{\alpha }:O_{K}^{N}\times O_{K}^{N}\to R$ defined by

(38) $$b_{\alpha }\left(x,y\right)=\sum_{i=1}^{N}{\mathrm{Tr}}_{K/Q}\left(\alpha x_{i}{\bar{y}}_{i}\right),$$

where α∈K∩R and ${\bar{y}}_{i}$ denotes the complex conjugate of $y_{i}$ if *K* is a CM field, and ${\bar{y}}_{i}=y_{i}$ if *K* is totally real. For $b_{\alpha }$ to be positive definite, α must be chosen to be totally positive, i.e., $\sigma_{i}\left(\alpha \right)>0$ for all real embeddings $\sigma_{i}:K\to R$ [53]. If α is chosen from the codifferent of *K* (i.e., $\alpha \in D_{K}^{-1}$), then $\mathrm{Tr}(\alpha x_{i}{\bar{y}}_{i})\in Z$ [53]. The pair $\left(\rho^{-1}\left(C\right),b_{\alpha }\right)$ thus forms a lattice of rank nN. The canonical embedding $\sigma^{N}$, defined componentwise as $\sigma^{N}:K^{N}\to R^{nN},\left(x_{1},\ldots ,x_{N}\right)\mapsto \left(\sigma \left(x_{1}\right),\ldots ,\sigma \left(x_{N}\right)\right)$, maps $\rho^{-1}\left(C\right)$ to a real lattice $\sigma^{N}\left(\rho^{-1}\left(C\right)\right)\subset R^{nN}$ of dimension nN.

This method of constructing lattices from linear codes is generally referred to as Construction A [46,49,53]. The original binary Construction A, proposed by Forney [71], used K=Q, $O_{K}=Z$, p=2 and typically α=1/2 or α=1. This binary version can be seen as a particular case of the cyclotomic field approach by Ebeling [47], which takes $K=Q(\zeta_{p})$, $O_{K}=Z\left[\zeta_{p}\right]$, and $p=(1-\zeta_{p})$. The generalization to CM fields and totally real number fields, particularly when p is totally ramified, has broadened the applicability of Construction A [49].

#### Generator and Gram Matrices for Construction A

The structure of the resulting lattice is explicitly defined by its generator and Gram matrices. Let n=[K:Q]. We consider the nN-dimensional lattice $\left(\rho^{-1}\left(C\right),b_{\alpha }\right)$. Let $\Delta =|d_{K}|$ be the absolute value of the discriminant of *K*.

**Proposition** **3** ([53])**.** *The following results hold:*

1. *The lattice $\left(\rho^{-1}\left(C\right),b_{\alpha }\right)$ has discriminant $\Delta^{N}p^{2f(N-k)}N{\left(\alpha \right)}^{N}$ and volume $\Delta^{\frac{N}{2}}p^{f(N-k)}N{\left(\alpha \right)}^{\frac{N}{2}}$.*
2. The dual lattice $\left(\rho^{-1}{\left(C\right)}^{*},b_{\alpha }\right)$ has discriminant $\Delta^{-N}p^{-2f(N-k)}N{\left(\alpha \right)}^{-N}$ and volume $\Delta^{\frac{-N}{2}}p^{-f(N-k)}N{\left(\alpha \right)}^{\frac{-N}{2}}$.
3. *The lattice $\left(\rho^{-1}\left(C^{⊥}\right),b_{\alpha }\right)$ has discriminant $\Delta^{N}p^{2fk}N{\left(\alpha \right)}^{N}$ and volume*
$$\Delta^{\frac{N}{2}}p^{fk}N{\left(\alpha \right)}^{\frac{N}{2}}.$$

Let $\left\{v_{1},\ldots ,v_{n}\right\}$ be a Z-basis for $O_{K}$ and let $\left\{\omega_{1},\ldots ,\omega_{n}\right\}$ be a Z-basis for p. Suppose C admits a generator matrix in the standard (systematic) form and let A be a matrix such that $\left[\begin{array}{cc} I_{k} & A\;(\mathrm{mod}\;p) \end{array}\right]$ is a generator matrix of C.

**Proposition** **4** ([53], Proposition 2.3)**.** *For K, a totally real number field of degree n with Galois group $G=\left\{\sigma_{1},\ldots ,\sigma_{n}\right\}$, a generator matrix for $\left(\rho^{-1}\left(C\right),b_{\alpha }\right)$ is given by*

(39) $$M_{C}=\left[\begin{array}{cc} I_{k}⊗M & A\tilde{⊗}M\\ 0_{n(N-k),nk} & I_{N-k}⊗M_{p} \end{array}\right]\left(I_{N}⊗D_{\alpha }\right),$$

*where $M={\left[\sigma_{j}\left(v_{i}\right)\right]}_{i,j=1,\ldots ,n}$, $M_{p}={\left[\sigma_{j}\left(\omega_{i}\right)\right]}_{i,j=1,\ldots ,n}$ are, respectively, generator matrices for $\left(O_{K}^{N},b_{1}\right)$ and $\left(p^{N},b_{1}\right)$, $D_{\alpha }$ is a diagonal matrix whose diagonal entries are $\sqrt{\sigma_{i}\left(\alpha \right)}$, i=1,…,n, and*

(40) $$A\tilde{⊗}M≜\left[\begin{array}{ccccccccccccc} B_{1,1} & \cdots & B_{1,n} & | & B_{2,1}\; & \cdots & B_{2,n} & | & \cdots & | & B_{N-k,1} & \cdots & B_{N-k,n} \end{array}\right],$$

*where $B_{j,i}=\sigma_{i}\left(A_{j}\right)⊗M_{i}$, in which $M_{i}$ and $A_{j}$ denote the ith and jth columns of the matrices M and A, respectively, for i=1,…,n and j=1,…,N−k. The operation of $\sigma_{i}$ is understood componentwise, for i=1,…,n. The Gram matrix $G_{C}=M_{C}M_{C}^{t}$ of $\left(\rho^{-1}\left(C\right),b_{\alpha }\right)$ is*

(41) $$G_{C}=\left[\begin{array}{cc} \mathrm{Tr}\left(\alpha \left(I_{k}+{\mathrm{AA}}^{t}\right)⊗M_{1}M_{1}^{t}\right) & \mathrm{Tr}\left(\alpha A⊗M_{1}M_{p,1}^{t}\right)\\ \mathrm{Tr}\left(\alpha A⊗M_{1}M_{p,1}^{t}\right) & \mathrm{Tr}\left(\alpha I_{N-k}⊗M_{p,1}M_{p,1}^{t}\right) \end{array}\right],$$

*where $\mathrm{Tr}={\mathrm{Tr}}_{K/Q}$ is taken componentwise, and $M_{1}$ and $M_{p,1}$ denote the first columns of the matrices M and $M_{p}$, respectively.*

When *K* is a CM number field, *n* is even and all embeddings of *K* into C are complex embeddings. Assume $\sigma_{i+1}$ is the conjugate of $\sigma_{i}$ for i=1,3,5,…,n−1.

**Proposition** **5** ([53], Proposition 2.6)**.** *Let K be a CM field with degree n and Galois group $G=\{\sigma_{1},\sigma_{2},\ldots ,\sigma_{n}\}$, where $\sigma_{i+1}$ is the conjugate of $\sigma_{i}$, for i=1,3,…,n−1. A generator matrix for $\left(\rho^{-1}\left(C\right),b_{\alpha }\right)$ is given by*

(42) $$M_{C}=\left[\begin{array}{cc} I_{k}⊗M & A\tilde{\tilde{⊗}}M\\ 0_{n(N-k),nk} & I_{N-k}⊗M_{p} \end{array}\right]\left(I_{N}⊗D_{\alpha }\right),$$

*where M is the generator matrix for the lattice $\left(O_{K},b_{1}\right)$ with $\det\left(M\right)=\Delta^{\frac{1}{2}}$ as follows:*

(43) $$M=\sqrt{2}\left[\begin{array}{cccccc} ℜ\sigma_{1}\left(v_{1}\right) & ℑ\sigma_{2}\left(v_{1}\right) & ℜ\sigma_{3}\left(v_{1}\right) & \ldots & ℜ\sigma_{n-1}\left(v_{1}\right) & ℑ\sigma_{n}\left(v_{1}\right)\\ \vdots & \vdots & \vdots & \ddots & \vdots & \vdots \\ ℜ\sigma_{1}\left(v_{n}\right) & ℑ\sigma_{2}\left(v_{n}\right) & ℜ\sigma_{3}\left(v_{n}\right) & \ldots & ℜ\sigma_{n-1}\left(v_{n}\right) & ℑ\sigma_{n}\left(v_{n}\right) \end{array}\right],$$

*and $M_{p}$ is the following generator matrix for $\left(p,b_{1}\right)$ and has determinant $\Delta^{\frac{1}{2}}p^{f}$*

(44) $$M_{p}=\sqrt{2}\left[\begin{array}{cccccc} ℜ\sigma_{1}\left(\omega_{1}\right) & ℑ\sigma_{2}\left(\omega_{1}\right) & ℜ\sigma_{3}\left(\omega_{1}\right) & \ldots & ℜ\sigma_{n-1}\left(\omega_{1}\right) & ℑ\sigma_{n}\left(\omega_{1}\right)\\ \vdots & \vdots & \vdots & \ddots & \vdots & \vdots \\ ℜ\sigma_{1}\left(\omega_{n}\right) & ℑ\sigma_{2}\left(\omega_{n}\right) & ℜ\sigma_{3}\left(\omega_{n}\right) & \ldots & ℜ\sigma_{n-1}\left(\omega_{n}\right) & ℑ\sigma_{n}\left(\omega_{n}\right) \end{array}\right].$$

*The matrix $D_{\alpha }$ is a diagonal matrix whose diagonal entries are $\sqrt{\sigma_{i}\left(\alpha \right)}$, i=1,…,n, A is a matrix such that $\left[\begin{array}{cc} I_{k} & A\;(\mathrm{mod}\;p) \end{array}\right]$ is a generator matrix of C and*

(45) $$A\tilde{\tilde{⊗}}M≜\left[\begin{array}{ccccccc} A_{1}^{\prime } & | & A_{2}^{\prime } & | & \ldots & | & A_{N-k}^{\prime } \end{array}\right],$$

*where $A_{j}^{\prime }=\left[\begin{array}{ccccccc} B_{1,j} & B_{1,j}^{\prime } & B_{3,j} & B_{3,j}^{\prime } & \ldots & B_{n-1,j} & B_{n-1,j}^{\prime } \end{array}\right]$, for j=1,…,N−k, in which $B_{i,j}=ℜ\sigma_{i}\left(A_{j}\right)⊗M_{i}+ℑ\sigma_{i}\left(A_{j}\right)⊗M_{i+1}$, $B_{i,j}^{\prime }=ℜ\sigma_{i}\left(A_{j}\right)⊗M_{i+1}-ℑ\sigma_{i}\left(A_{j}\right)⊗M_{i}$ with $A_{j}$ and $M_{i}$ as the jth and ith columns of A and M, respectively, for j=1,…,N−k and i=1,…,n. We understand ℜ and ℑ componentwise.*

**Remark** **1** ([53], Remark 2.7)**.** *Let $v={\left(v_{1},v_{2},\ldots ,v_{n}\right)}^{t}$ and $v_{j}^{\prime }=A_{j}⊗v$, for j=1,…,N−k, then*

(46) $$A\tilde{\tilde{⊗}}M=\sqrt{2}\left[\begin{array}{ccccccc} V_{1}^{\prime } & | & V_{2}^{\prime } & | & \ldots & | & V_{N-k}^{\prime } \end{array}\right],$$

*where $V_{j}^{\prime }=\left[\begin{array}{ccccc} ℜ\sigma_{1}\left(v_{j}^{\prime }\right) & ℑ\sigma_{2}\left(v_{j}^{\prime }\right) & \ldots & ℜ\sigma_{n-1}\left(v_{j}^{\prime }\right) & ℑ\sigma_{n}\left(v_{j}^{\prime }\right) \end{array}\right]$, for j=1,…,N−k. When p is totally ramified, the entries of A(modp) are in $F_{p}$ and hence $A\tilde{\tilde{⊗}}M=A⊗M$. In this case, the generator matrix of $\left(\rho^{-1}\left(C\right),b_{1}\right)$ coincides with the one obtained in [49], Proposition 1.*

**Proposition** **6** ([53], Proposition 2.8)**.** *The Gram matrix $G_{C}=M_{C}M_{C}^{t}$ of $\left(\rho^{-1}\left(C\right),b_{\alpha }\right)$ is*

(47) $$G_{C}=\left[\begin{array}{cc} \mathrm{Tr}\left(\alpha \left(I_{k}+AA^{†}\right)⊗vv^{†}\right) & \mathrm{Tr}\left(\alpha A⊗(v\omega^{†})\right)\\ \mathrm{Tr}\left(\alpha A^{t}⊗\left(\bar{\omega }v^{t}\right)\right) & \mathrm{Tr}\left(\alpha I_{N-k}⊗\omega \omega^{†}\right) \end{array}\right],$$

*where $\mathrm{Tr}={\mathrm{Tr}}_{K/Q}$ is taken componentwise, $\omega ={\left(\omega_{1},\omega_{2},\ldots ,\omega_{n}\right)}^{t}$ and $v^{†}={\bar{v}}^{t}$ is the conjugate transpose of v. Similarly, $A^{†}={\bar{A}}^{t}$ and $\omega^{†}={\bar{\omega }}^{t}$.*

Two particular cases of the above construction method, when α=1/p or α=1/2p for *K* a real quadratic field with p inert and *K* an imaginary quadratic field with p totally ramified, have been discussed in [53].

### 4.3. Known Modular/Unimodular Constructions

Self-dual codes play a crucial role in constructing modular and unimodular lattices, a property that makes them particularly interesting for communication theory due to their well-behaved characteristics.

**Proposition** **7** ([53], Proposition 2.9)**.** *If C is not self-orthogonal, i.e., if $C⊄C^{⊥}$, then $\left(\rho^{-1}\left(C\right),b_{\alpha }\right)$ is not an integral lattice for any $\alpha \in p^{-1}\cap Q$ when K is totally real or when K is a CM field and p is totally ramified.*

This proposition justifies why we consider self-orthogonal codes in the construction of modular lattices, which are of great interest in information security. Past work has extensively explored modular lattice constructions from quadratic number fields [53]:

- **Totally Real Quadratic Fields:** Let $K=Q(\sqrt{d})$ be a totally real quadratic field, where *d* is a positive squarefree integer. Its Galois group is $\left\{\sigma_{1},\sigma_{2}\right\}$. The discriminant $d_{K}$ is *d* for d≡1(mod4), and 4d for d≡2,3(mod4). For a prime p∈Z inert in *K*, and a linear code C over $F_{p^{2}}$, if C is self-orthogonal, then $\left(\rho^{-1}\left(C\right),b_{\alpha }\right)$ is an integral lattice. Furthermore, if C is self-dual, then it forms a *d*-modular lattice with α=1/p (for d≡1(mod4)) or α=1/2p (for d≡2,3(mod4)).
- **Imaginary Quadratic Fields (CM Fields):** Let $K=Q(\sqrt{-d})$ be an imaginary quadratic field (*d* a positive squarefree integer). Its Galois group is $\left\{\sigma_{1},\sigma_{2}\right\}$ where $\sigma_{1}$ is the identity and $\sigma_{2}$ is complex conjugation. The discriminant Δ is 4d for d≡1,2(mod4), and *d* for d≡3(mod4). For a prime p∈Z totally ramified in *K*, and a linear code C over $F_{p}$, if C is self-orthogonal, then $\left(\rho^{-1}\left(C\right),b_{\alpha }\right)$ is integral. If C is self-dual, it produces unimodular lattices for α=1/p (for d≡3(mod4)) or α=1/2p (for d≡1,2(mod4)).

These results demonstrate how algebraic properties (type of field, prime factorization behavior) directly influence the modularity and integrality of constructed lattices.

### 4.4. Classical Families Used for Wiretap Codes

The development of lattice codes for wiretap channels has relied heavily on classical families of self-dual codes, which enable the construction of modular and unimodular lattices. These families are critical for balancing coding gain and secrecy performance.

**Definition** **14.** *A linear code $C\subset F_{q}^{N}$ of dimension k over a finite field $F_{q}$ (where q is a prime power) has a dual code $C^{⊥}=\left\{x\in F_{q}^{N}\;|\;x\cdot y=\sum_{i=1}^{N}x_{i}y_{i}=0\;\;\mathrm{for}\;\;\mathrm{all}\;\;y\in C\right\}$. C is self-orthogonal if $C\subset C^{⊥}$, and is self-dual if $C=C^{⊥}$.*

It is well known for the binary Construction A that $C\subset F_{2}^{N}$ is self-dual if and only if $\left(\rho^{-1}\left(C\right),b_{\frac{1}{2}}\right)$ is unimodular [46,47]. More generally, for $K=Q(\zeta_{p})$, if $C\subset F_{p}^{N}$ is self-dual, then $\left(\rho^{-1}\left(C\right),b_{\frac{1}{p}}\right)$ is unimodular [47]. The converse of this statement is proved in [53] for totally real number fields and CM fields with a totally ramified prime.

Self-dual codes thus provide a systematic way to obtain modular lattices. Specific examples include the following:

- **2-Modular Lattices:** Constructed from self-dual codes over $F_{3}$ in imaginary quadratic fields like $K=Q(\sqrt{-2})$ [72].
- **3-Modular Lattices:** Constructed from self-dual codes over $F_{4}$ in fields like $K=Q(\zeta_{3})$ [73].
- **Other Modular Lattices:** Quadratic fields ($Q(\sqrt{-7})$, Q(i)) and specific cyclotomic fields ($Q(\zeta_{3})$) have been used with self-dual codes to construct various modular lattices [74].

More generalized versions of Construction A have also been introduced, where the ring of integers $O_{K}$ is replaced by any lattice $L\subset R^{n}$, further expanding the scope of lattice constructions [75].

#### Self-Dual Codes for 5-Modular Lattices

For our construction of 5-modular lattices, we need a family of self-dual codes over $F_{5}$. These codes exist if and only if their length is even [76]. A key property for a codeword u in a self-orthogonal code C over $F_{5}$, if it contains *i* 0’s, *j*{±1}’s and *k*{±2}’s (so that the Hamming weight of u is j+k), then u·u=0 implies

(48) j≡k(mod5).

Equation (48) also implies that a codeword in a self-orthogonal code cannot have weight 1 or 3, although all other weights can occur [76]. Well-known 5-ary self-dual codes include [76]:

- $C_{2}$: The [2,1,2] code consisting of codewords {(0,0),(1,2),(2,−1),(−2,1),(−1,−2)}, with generator matrix $\left[1\;2\right]$.
- $F_{N}$: The [N,N/2,4] code, for N=6,8,10,…, with a specific generator matrix structure
  (49) $$\left[\begin{array}{cccccccccccccc} 1 & -1 & 2 & 2 & 0 & 0 & 0 & 0 & 0 & \ldots & 0 & 0 & 0 & 0\\ 0 & 0 & 1 & -1 & 2 & 2 & 0 & 0 & 0 & \ldots & 0 & 0 & 0 & 0\\ 0 & 0 & 0 & 0 & 1 & -1 & 2 & 2 & 0 & \ldots & 0 & 0 & 0 & 0\\ \vdots & \vdots & \vdots & \vdots & \vdots & \vdots & \vdots & \vdots & \vdots & \ddots & \vdots & \vdots & \vdots & \vdots \\ 0 & 0 & 0 & 0 & 0 & 0 & 0 & 0 & 0 & \ldots & 1 & -1 & 2 & 2\\ 2 & 2 & 0 & 0 & 0 & 0 & 0 & 0 & 0 & \ldots & 0 & 0 & 1 & -1 \end{array}\right].$$
  Exceptional cases like $F_{6}$ and $F_{8}$ have more convenient generator matrix forms. For $F_{6}$, it is more convenient to use the generator matrix
  (50) $$\left[\begin{array}{cccccc} 1 & 0 & 1 & -1 & -1 & 1\\ 1 & 1 & 0 & 1 & -1 & -1\\ 1 & -1 & 1 & 0 & 1 & -1 \end{array}\right].$$
  For $F_{8}$, it is convenient to use the generator matrix $\left[\begin{array}{ccc} I_{4} & | & H_{4} \end{array}\right]$, where $I_{n}$ denotes an n×n identity matrix and $H_{4}$ is a Hadamard matrix
  (51) $$H_{4}=\left[\begin{array}{cccc} 1 & 1 & 1 & 1\\ 1 & -1 & 1 & -1\\ 1 & 1 & -1 & -1\\ 1 & -1 & -1 & 1 \end{array}\right].$$
- $L_{N}$: The Hadamard codes, denoted by $L_{N}$, contain three obvious classes of self-dual codes that can be obtained from Hadamard matrices. Let $H_{t}$ denote an arbitrary Hadamard matrix of order *t*.
  - For N≡0(mod20), let $L_{N}$ be the code generated by the rows of $H_{N}$.
  - For N≡8(mod40), let $L_{N}$ be the code with generator matrix $\left[\begin{array}{ccc} I_{N/2} & | & H_{N/2} \end{array}\right]$.
  - For N≡32(mod40), let $L_{N}$ have generator matrix $\left[\begin{array}{ccc} 2I_{N/2} & | & H_{N/2} \end{array}\right]$. For example, there is a unique code $L_{8}$ which is equivalent to $F_{8}$.
- $Q_{N}$ and $Q_{N}^{\prime }$: The quadratic residue codes $Q_{N}$ and $Q_{N}^{\prime }$ differ from the Hadamard codes only in that the diagonal entries of the Hadamard matrices are altered [76].
  - When N≡0 or 12(mod20) and N−1=q is a prime power, $Q_{N}$ is the code with generator matrix $\left(m_{i,j}\right)$, where the rows and columns are labeled ∞,0,l,…,q−1, and $m_{i,i}=\sqrt{-q}$ for all *i*, $m_{\infty ,i}=1$ and $m_{i,\infty }=-1$. For i≥0, $m_{i,j}=1$ if j−i is a square in $F_{q}$, and $m_{i,j}=-1$ if j−i is a nonsquare (i,j>0,i≠j). Then, $Q_{N}$ is the usual self-dual extended quadratic residue code (see, for example, [77,78]). The first four of these codes are $Q_{12}=\left[12,6,6\right]$, $Q_{20}=\left[20,10,8\right]$, $Q_{32}=\left[32,16,10\right]$ and $Q_{60}=\left[60,30,d\leq 18\right]$.
  - A *conference matrix* $B_{N}$ is a real N×N matrix with diagonal entries 0 and other entries ±1 which satisfies $BB^{t}=\left(n-1\right)I_{N}$. For N≡6(mod10), let $Q_{N}$ be the self-dual code generated by the rows of a conference matrix $B_{N}$, if one exists. For example, $Q_{6}≅F_{6}$. A more interesting example is obtained from $B_{16}$, which gives a [16,8,7] code $Q_{16}$ with generator matrix
    (52) $$\left[\begin{array}{cccc} B_{4} & H_{4} & H_{4} & H_{4}\\ -H_{4} & B_{4} & H_{4} & -H_{4} \end{array}\right],$$
    where $H_{4}$ is given in (51) and
    (53) $$B_{4}=\left[\begin{array}{cccc} 0 & 1 & 1 & 1\\ -1 & 0 & 1 & -1\\ -1 & -1 & 0 & 1\\ -1 & 1 & -1 & 0 \end{array}\right].$$
  - For N≡12(mod20), let $Q_{N}^{\prime }$ be the code generated by the rows of $B_{N}+2I_{N}$, where $B_{N}$ is a skew-symmetric conference matrix.
  - For N≡0(mod20), let $Q_{N}^{\prime }$ be generated by $\left[\begin{array}{ccc} I_{N/2} & | & B_{N/2} \end{array}\right]$, where $B_{N/2}$ is any conference matrix. For example, using the Paley matrix $B_{20}$ produces a [40,20,13] code $Q_{40}^{\prime }$ [76].
  - For N≡32(mod40), let $Q_{N}^{\prime \prime }$ be generated by $\left[\begin{array}{ccc} I_{N/2} & | & B_{N/2}+2I_{N/2} \end{array}\right]$, where $B_{N/2}$ is skew-symmetric.
  - For N≡4(mod20), let $Q_{N}$, be generated by $\left[\begin{array}{ccc} 2I_{N/2} & | & B_{N/2} \end{array}\right]$, where $B_{N/2}$ is any conference matrix. For example, using the Paley matrix $B_{12}$ produces a [24,12,9] code $Q_{24}$.
  - Finally, for N≡16(mod40), let $Q_{N}^{\prime }$ generated by $\left[\begin{array}{ccc} 2I_{N/2} & | & B_{N/2}+2I_{N/2} \end{array}\right]$, where $B_{N/2}$ is skew-symmetric.

## 5. Cyclotomic Fields and Modular Lattices

This section bridges the theoretical foundations of algebraic number theory with the practical construction of modular lattices for physical-layer security. We specifically focus on cyclotomic fields, which offer a rich algebraic structure for generating lattices with desirable properties. This survey consolidates classical and modern results on cyclotomic lattices, analyzes the conditions for their modularity, and identifies existing limitations, thereby setting the stage for our novel contributions in the next section.

### 5.1. Classical Cyclotomic Lattices

Cyclotomic fields, due to their well-understood algebraic properties and monogenicity (as discussed in Section 3.2), have been a fertile ground for lattice constructions, particularly in coding theory. Many interesting lattices can be constructed as ideal lattices over cyclotomic fields $K=Q(\zeta_{m})$. For instance, root lattices like $E_{8}$ (for m=15,20,24), the Coxeter–Todd lattice $K_{12}$ (for m=21), and the Leech lattice $\Lambda_{24}$ (for m=35,39,52,56,84) have been realized as cyclotomic lattices [79].

Constructions of modular ideal lattices over specific cyclotomic fields $Q(\zeta_{m})$ have been extensively studied. For example, for $m=p^{r}$ (*p* prime, r≥1), a construction results in an even, *p*-modular ideal lattice of trace type over $K=Q(\zeta_{p^{r}})$, with a specified rank and determinant [79], Proposition 1. Similar constructions exist for $m=p^{r}q^{s}$ [79], Proposition 2.

**Theorem** **10** ([79], Theorem 1)**.** *There exists a modular ideal lattice over $K=Q(\zeta_{m})$ if and only if m is not a power of a prime p with p≡1(mod4).*

The authors of [79] have also characterized the cyclotomic fields for which there exists a modular ideal lattice of trace type.

**Definition** **15.** *Let p be a prime divisor of m. Then, p is a norm of m if we have $D_{K}=IJ\bar{J}$ for some integral $O_{K}$-ideals I and J such that J is above p and I is prime to p. Let $m^{\prime }$ be a divisor of m. Then, $m^{\prime }$ is a norm of m if all the prime divisors of $m^{\prime }$ are norms of m.*

**Theorem** **11** ([79], Theorem 2)**.** *Let ℓ=1 or a prime number p, with p≡1(mod4). Let $m=\ell^{r}m^{\prime }$, with $m^{\prime }$ prime to ℓ. Then, there exists an ℓ-modular ideal lattice of trace type if and only if $m^{\prime }$ is a norm of m.*

For ℓ=1 (i.e., unimodular lattices), if *m* is a norm of *m*, then $L_{m}^{1}\left(J\right)$ (constructed with $I=J^{-1}$ and $b\left(x,y\right)=\mathrm{Tr}\left(x\bar{y}\right)$) is a unimodular ideal lattice of trace type, and even if *m* is not a power of 2 [79], Proposition 5. For ℓ=p (*p* prime, p≢1(mod4)), similar constructions yield *p*-modular ideal lattices of trace type [79], Proposition 6.

### 5.2. Conditions for Modularity

The modularity property is highly desirable for lattice codes in communication, as it ensures certain symmetries and favorable characteristics for secrecy metrics. Understanding the algebraic conditions that lead to modularity is therefore critical.

A lattice (Λ,b) is *d*-modular if it is integral and its dual $\Lambda^{*}$ is similar to Λ scaled by $\sqrt{d}$, i.e., $\Lambda^{*}=\sqrt{d}\Lambda$. For Construction A over number fields, the modularity conditions are deeply intertwined with the properties of the underlying number field *K*, the choice of the element α, and the characteristics of the linear code C.

The key conditions for modularity in Construction A include the following:

- **Self-Dual Codes:** As shown in Proposition 2.9 [53], self-orthogonal codes are a prerequisite for integrality. Specifically, for Construction A over $Q(\zeta_{p})$, a self-dual code C over $F_{p}$ leads to a unimodular lattice $\left(\rho^{-1}\left(C\right),b_{1/p}\right)$ [47].
- **Discriminant and Trace Relations:** The determinant of the lattice, its dual, and the properties of the trace form (${\mathrm{Tr}}_{K/Q}$) play a direct role in determining modularity. The relation $\mathrm{vol}\left(\Lambda \right)=d^{n/4}$ for a *d*-modular lattice is a key property.
- **Choice of α:** The specific choice of α∈K in the bilinear form $b_{\alpha }$ is critical. For instance, α=1/p or α=1/2p are common choices to achieve unimodularity or modularity over quadratic fields [53].

These algebraic conditions dictate whether the constructed lattice will possess the desired modularity, which directly influences its suitability for specific wireless communication scenarios.

### 5.3. Existing Results and Limitations

While Construction A over number fields is a powerful tool for designing lattices, achieving modularity for specific field types and parameters is not always straightforward. Prior research has identified both successful constructions and inherent limitations, often tied to the specific algebraic properties of the chosen number fields.

Notable successes in constructing modular and unimodular lattices from cyclotomic fields via Construction A include the following.

**Proposition** **8** ([47], Section 5.2, [48], Example 1)**.** *For an odd prime p, let $K=Q(\zeta_{p})$ be a CM field with ring of integers $O_{K}=Z\left[\zeta_{p}\right]$. Taking $p=(1-\zeta_{p})$ and defining the bilinear form $b_{1/p}\left(x,y\right)=\sum_{i=1}^{N}{\mathrm{Tr}}_{K/Q}\left(x_{i}{\bar{y}}_{i}/p\right)$, if the code $C\subset C^{⊥}$ over $F_{p}$, then $\left(\rho^{-1}\left(C\right),b_{1/p}\right)$ yields an even integral lattice of rank N(p−1). Furthermore, if C is self-dual, the resulting lattice is an even unimodular lattice.*

**Proposition** **9** ([49], Corollary 2)**.** *Let $K^{+}=Q\left(\zeta_{p}+\zeta_{p}^{-1}\right)$ be a totally real subfield. For a code $C\subset F_{p}^{N}$ with $C\subset C^{⊥}$, the lattice $\left(\rho^{-1}\left(C\right),b_{\alpha }\right)$ formed with α=1/p is an integral lattice of rank N(p−1)/2. If C is self-dual, this construction produces an odd unimodular lattice.*

However, these successful constructions are often contrasted with scenarios where achieving modularity proves challenging, indicating inherent constraints in the design space. For instance, in this paper, we demonstrate a counter example where, for $K=Q(\zeta_{6})$, a 3-modular lattice cannot be constructed via Construction A, despite initial conditions suggesting its feasibility. Similarly, for $K=Q\left(\zeta_{4}\right)=Q\left(i\right)$ and prime p=5, our result shows that a 5-modular lattice is not obtained from Construction A, even with an integral code over $F_{5}$.

These examples highlight that achieving modularity is not universally guaranteed. Furthermore, the literature has generally not addressed the specific case of cyclotomic fields $Q(\zeta_{p^{r}})$ and $Q(\zeta_{p^{r}}+\zeta_{p^{r}}^{-1})$ for ramification degree r>1, nor general cyclotomic fields $Q(\zeta_{n})$ where *n* is not a prime power. The observed difficulties in achieving modularity under specific conditions, coupled with these unaddressed cases, represent significant gaps and limitations in existing Construction A frameworks for cyclotomic fields. Our work, therefore, aims to directly address these limitations by rigorously establishing new existence and non-existence results for *p*-modular lattices derived from these specific types of cyclotomic fields.

## 6. Newly Proposed p-Modular Lattices for p≡1(mod4)

This section reviews the newly proposed family of *p*-modular lattices in [80] specifically constructed from cyclotomic number fields, addressing some of the limitations identified in the existing literature (Section 5.3). Following [80], we review the construction methodology, characterize the key algebraic and geometric properties of these lattices, and rigorously establish non-existence results for certain parameter choices. These findings provide critical design principles and insights into the suitability of these new lattices for physical-layer security applications.

The importance of cyclotomic number fields in the construction of modular lattices for wireless communication cannot be overstated. Their well-defined algebraic properties and monogenicity make them ideal candidates for systematic lattice design. In this section, we present a new framework based on Construction A and cyclotomic number fields, yielding a family of *p*-modular lattices where p≡1(mod4).

A foundational result in algebraic number theory is the Kronecker–Weber theorem, which highlights the pervasive nature of cyclotomic fields in number theory. Every cyclotomic field is an Abelian extension of the rational number field Q. The Kronecker–Weber theorem, first announced by Kronecker in 1853, provides a powerful converse.

**Theorem** **12** (Kronecker–Weber [81,82])**.** *Every finite Abelian extension of Q lies in a cyclotomic field $Q(\zeta_{m})$ for some integer m.*

In other words, any algebraic integer in a number field whose Galois group is Abelian can be expressed as a sum of roots of unity with rational coefficients. Let $Q_{m}=Q\left(e^{2\pi i/m}\right)$. We can assume that m≢2(mod4), because if m≡2(mod4) with $m=2m_{0}$, then it is easy to check that $e^{-2\pi i/m_{0}}$ is a primitive *m*th root of unity, and hence $Q_{m}=Q_{m_{0}}$. The Kronecker–Weber theorem motivates the following definition.

**Definition** **16** (Conductor)**.** *Let L/Q be a finite Abelian extension. A positive integer m is a defining modulus or an admissible modulus of L if $L\subset Q_{m}$. Such an m exists by the Kronecker–Weber theorem. The conductor of L, $f_{L}$, is the smallest admissible modulus of L.*

**Example** **2** ([83])**.** *Let $L=Q(\zeta_{m}+\zeta_{m}^{-1})$ be the maximal real subfield of $Q_{m}$. For m≥5, its conductor $f_{L}=m$. If m=3,4, then L=Q and $f_{L}=1$. As another case, consider $L=Q(\sqrt{d})$, where d is a squarefree integer, |d|>1. Then, its conductor is given by*

(54) $$f_{L}=\left|d_{L}\right|=\left\{\begin{array}{cc} |d|, & if\;\;d\equiv 1\;\;\;(\mathrm{mod}\;4),\\ |4d|, & if\;\;d\equiv 2,3\;\;\;(\mathrm{mod}\;4). \end{array}\right.$$

*If $L=Q_{p}$ (p an odd prime), then using (26), $d_{L}={\left(-1\right)}^{\frac{p-1}{2}}p^{p-2}$ is the square of an integer in $O_{L}$; thus, $Q\left(\sqrt{{\left(-1\right)}^{\frac{p-1}{2}}p}\right)\subset Q_{p}$. It follows that for a prime p:*

(55) $$Q\left(\sqrt{p}\right)\subset \left\{\begin{array}{cc} Q_{p}, & if\;\;p\equiv 1\;\;\;(\mathrm{mod}\;4),\\ Q_{4p}, & if\;\;p\equiv 3\;\;\;(\mathrm{mod}\;4),\\ Q_{8}, & if\;\;p=2. \end{array}\right.$$

*Moreover, if $d=\pm 2^{\nu }p_{1}p_{2}\cdots p_{r}$ is squarefree, then $Q\left(\sqrt{d}\right)\subset Q_{4d}$.*

### 6.1. Construction: Generalized Construction A from $Q(\zeta_{p})$

Our primary construction leverages the generalized Construction A framework (Section 4.2) applied to cyclotomic number fields $Q(\zeta_{p})$ for primes p≡1(mod4). This specific choice of number field allows for the creation of *p*-modular lattices with well-defined properties.

**Theorem** **13.** *Let $K=Q(\zeta_{p})$ where p is an odd prime and p≡1(mod4), with the ring of integers $O_{K}=Z\left[\zeta_{p}\right]$. Then, K is a CM field and the prime p totally ramifies in K as $pO_{K}=P^{p-1}$, with residue field $O_{K}/P≅F_{p}$, where $P=pO_{K}+\left(1-\zeta_{p}\right)O_{K}$. Let $C\subset F_{p}^{N}$ be an (N,k) self-dual code over $F_{p}$. Then, $\left(\rho^{-1}\left(C\right),b_{\alpha }\right)$ with $b_{\alpha }\left(x,y\right)=\sum_{i=1}^{N}{\mathrm{Tr}}_{K/Q}\left(\alpha x_{i}{\bar{y}}_{i}\right)$ and $\alpha =\frac{1}{\sqrt{p}}$, is a p-modular lattice.*

**Proof.**  See Appendix A.1. □

**Remark** **2.** *For $K=Q(\zeta_{p})$, the p−1 embeddings $\sigma_{1},\ldots ,\sigma_{p-1}:K\to C$ are given by*

(56) $$\sigma_{r}\left(\zeta_{p}\right)=\zeta^{r},\;r=1,\ldots ,p-1.$$

*Then, the trace of an element γ∈K, $\gamma =a_{0}+a_{1}\zeta_{p}+\ldots +a_{p-2}\zeta_{p}^{p-2}$, $a_{i}\in Q$, is easily computed to be [47], p. 122*

(57) $${\mathrm{Tr}}_{K/Q}\left(\gamma \right)=\left(p-1\right)a_{0}-a_{1}-a_{2}-\cdots -a_{p-2}.$$

*Let $P=(1-\zeta_{p})$ be the principal ideal of $O_{K}=Z\left[\zeta_{p}\right]$ generated by the element $1-\zeta_{p}$ in $O_{K}$. Then, P∩Z=pZ and for any x∈P, ${\mathrm{Tr}}_{K/Q}\left(x\right)\in pZ$ [47], p. 122. The mapping $\rho :O_{K}\to F_{p}$ sending $\gamma =a_{0}+a_{1}\zeta_{p}+\cdots +a_{p-2}\zeta_{p}^{p-2}$, $a_{i}\in Z$, to $\rho \left(\gamma \right)=a_{0}+a_{1}+\cdots +a_{p-2}\;\left(\mathrm{mod}\;p\right)$ is an additive homomorphism and the kernel of this homomorphism is equal to P. This shows that the mapping ρ can be considered as the reduction modP [47], p. 123. The vectors*

(58) $$1-\zeta_{p},\zeta_{p}-\zeta_{p}^{2},\zeta_{p}^{2}-\zeta_{p}^{3},\ldots ,\zeta^{p-2}-\zeta^{p-1}$$

*form a Z-basis for P [47], p. 126.*

**Remark** **3.** *In order to use Theorem 13, we need to express $\sqrt{p}$ in terms of the Z-basis of $Z[\zeta_{p}]$. To this end, we use quadratic Gauss sums. A quadratic Gauss sum can be interpreted as a linear combination of the values of the complex exponential function with coefficients given by a quadratic character (for a general character, one obtains a more general Gauss sum) [84], pp. 70–76. Let p be an odd prime number and a an integer. Then, the Gauss sum (modp), g(a;p), is the sum of the pth roots of unity*

(59) $$g\left(a;p\right)=\sum_{k=0}^{p-1}e^{2\pi iak^{2}/p}=\sum_{k=0}^{p-1}\zeta_{p}^{ak^{2}}.$$

*If a is not divisible by p, an alternative expression for the Gauss sum is*

(60) $$G\left(a,\chi \right)=\sum_{k=0}^{p-1}\chi \left(k\right)e^{2\pi iak/p},$$

*where $\chi \left(k\right)=\left(\frac{k}{p}\right)$ is the Legendre symbol, which is a quadratic character (modp). Putting a general character χ in place of the Legendre symbol defines the Gauss sum G(χ). The value of the Gauss sum is an algebraic integer in $Q(\zeta_{p})$. The evaluation of the Gauss sum can be reduced to the case a=1 as follows:*

(61) $$g\left(a;p\right)=\left(\frac{a}{p}\right)g\left(1;p\right).$$

*We also have the following useful result [84], p. 75*

(62) $$g\left(1;p\right)=\sum_{k=0}^{p-1}e^{2\pi ik^{2}/p}=\left\{\begin{array}{ccc} \sqrt{p} & \; & p\equiv 1\;\;(\mathrm{mod}\;\;\;4),\\ i\sqrt{p} & \; & p\equiv 3\;\;(\mathrm{mod}\;\;\;4). \end{array}\right.$$

The main reason for applying the canonical embedding on algebraic lattices is the embedding of their corresponding lattices, into the real space $R^{n}$ for some *n*. Theorem 13 does not guarantee that its introduced lattice is embedded in $R^{N(p-1)}$, because the element α is not necessarily totally positive and some of $\sqrt{\sigma_{i}\left(\alpha \right)}$’s may be purely imaginary numbers. This issue, particularly for p=5, is addressed in the following proposition.

**Proposition** **10.** Let p=5 and $\rho^{-1}\left(C\right)$ be the obtained lattice in Theorem 13. Define $\Gamma_{C}=\sigma^{N}\left(\rho^{-1}\left(C\right)\right)$, where $\sigma^{N}$ is the canonical embedding which has been applied componentwise over $O_{K}^{N}$; that is, $\sigma^{N}\left(x_{1},\ldots ,x_{N}\right)=\left(\sigma \left(x_{1}\right),\ldots ,\sigma \left(x_{N}\right)\right)$, for $\left(x_{1},\ldots ,x_{N}\right)\in O_{K}^{N}$ and $\sigma =(\sigma_{1},\ldots ,\sigma_{4})$. Then, $\Gamma_{C}$ is a Z-lattice in $R^{2N}\times R^{*2N}$, where $R^{*}$ is the set of purely imaginary numbers.

**Proof.**  See Appendix A.2. □

Using Theorem 13, we find a new family of 5-modular lattices which is applicable in information security. We need a family of self dual codes over $F_{5}$, which is provided in [76].

**Example** **3.** *Let p=5 and $K=Q(\zeta_{5})$, with $\rho :Z{\left[\zeta_{5}\right]}^{2}\to F_{5}^{2}$ given in Remark 2. The degree of K/Q is 4, and the four embeddings of K are $\sigma_{1}$, which is the identity, $\sigma_{2}$, which is the conjugate of $\sigma_{1}$ and maps $\zeta_{5}$ to $\zeta_{5}^{4}$, $\sigma_{3}$, which maps $\zeta_{5}$ to $\zeta_{5}^{2}$, and $\sigma_{4}$, which is the conjugate of $\sigma_{3}$ and maps $\zeta_{5}$ to $\zeta_{5}^{3}$. Consider the self dual code $C=C_{2}$ of length 2 over $F_{5}$ as in the above, with generator matrix $\left[\begin{array}{cc} I & A\;(\mathrm{mod}\;5) \end{array}\right]$ and $A\;\left(\mathrm{mod}\;5\right)=\left[\begin{array}{c} 2 \end{array}\right]$. Using the mapping ρ is Remark 2, we can take 2 to be the preimage of 2 and we have $A=\left[\begin{array}{c} 2 \end{array}\right]$. We next compute a generator matrix for the lattice $\rho^{-1}\left(C\right)$ explicitly using the discussion from Section 4. We choose the basis $\left\{v_{1}=1,v_{2}=\zeta_{5},v_{3}=\zeta_{5}^{2},v_{4}=\zeta_{5}^{3}\right\}$ for $O_{K}$, and it follows that the generator matrix for the lattice $O_{K}$ together with the trace form $\langle x,y\rangle ={\mathrm{Tr}}_{K/Q}\left(x\bar{y}\right)$, $x,y\in O_{K}$, is*

(63) $$M=\sqrt{2}\left[\begin{array}{cccc} ℜ\sigma_{1}\left(1\right) & ℑ\sigma_{2}\left(1\right) & ℜ\sigma_{3}\left(1\right) & ℑ\sigma_{4}\left(1\right)\\ ℜ\sigma_{1}\left(\zeta_{5}\right) & ℑ\sigma_{2}\left(\zeta_{5}\right) & ℜ\sigma_{3}\left(\zeta_{5}\right) & ℑ\sigma_{4}\left(\zeta_{5}\right)\\ ℜ\sigma_{1}\left(\zeta_{5}^{2}\right) & ℑ\sigma_{2}\left(\zeta_{5}^{2}\right) & ℜ\sigma_{3}\left(\zeta_{5}^{2}\right) & ℑ\sigma_{4}\left(\zeta_{5}^{2}\right)\\ ℜ\sigma_{1}\left(\zeta_{5}^{3}\right) & ℑ\sigma_{2}\left(\zeta_{5}^{3}\right) & ℜ\sigma_{3}\left(\zeta_{5}^{3}\right) & ℑ\sigma_{4}\left(\zeta_{5}^{3}\right) \end{array}\right]=\sqrt{2}\left[\begin{array}{cccc} 1 & 0 & 1 & 0\\ ℜ\zeta_{5} & ℑ\zeta_{5}^{4} & ℜ\zeta_{5}^{2} & ℑ\zeta_{5}^{3}\\ ℜ\zeta_{5}^{2} & ℑ\zeta_{5}^{3} & ℜ\zeta_{5}^{4} & ℑ\zeta_{5}\\ ℜ\zeta_{5}^{3} & ℑ\zeta_{5}^{2} & ℜ\zeta_{5} & ℑ\zeta_{5}^{4} \end{array}\right].$$

*It should be noted that*

$$\begin{array}{ccc} ℜ\zeta_{5}=ℜ\zeta_{5}^{4}=\cos\left(\frac{2\pi }{5}\right)=\frac{-1+\sqrt{5}}{4}, & \; & ℜ\zeta_{5}^{2}=ℜ\zeta_{5}^{3}=\cos\left(\frac{4\pi }{5}\right)=\frac{-1-\sqrt{5}}{4}, \end{array}$$

$$\begin{array}{ccc} ℑ\zeta_{5}=\frac{1}{4}\sqrt{10+2\sqrt{5}}, & \; & ℑ\zeta_{5}^{4}=\frac{-1}{4}\sqrt{10+2\sqrt{5}},\\ ℑ\zeta_{5}^{2}=\frac{1}{4}\sqrt{10-2\sqrt{5}}, & \; & ℑ\zeta_{5}^{3}=\frac{-1}{4}\sqrt{10-2\sqrt{5}}. \end{array}$$

*Using Proposition 5, a generator matrix for $\left(\rho^{-1}\left(C\right),b_{\alpha }\right)$, with $\alpha =1/\sqrt{5}$, is*

(64) $$M_{C}=\left[\begin{array}{cc} M & A⊗M\\ 0_{4\times 4} & M_{p} \end{array}\right]\left(I_{2}⊗D_{\alpha }\right),$$

*where $M_{p}$ is obtained using the Z-basis $\{\omega_{1}=1-\zeta_{5},\omega_{2}=\zeta_{5}-\zeta_{5}^{2},\omega_{3}=\zeta_{5}^{2}-\zeta_{5}^{3},$$\omega_{4}=\zeta_{5}^{3}-\zeta_{5}^{4}\}$ for P as follows:*

(65) $$\begin{array}{ccc} M_{p} & = & \sqrt{2}\left[\begin{array}{cccc} ℜ\sigma_{1}\left(\omega_{1}\right) & ℑ\sigma_{2}\left(\omega_{1}\right) & ℜ\sigma_{3}\left(\omega_{1}\right) & ℑ\sigma_{4}\left(\omega_{1}\right)\\ ℜ\sigma_{1}\left(\omega_{2}\right) & ℑ\sigma_{2}\left(\omega_{2}\right) & ℜ\sigma_{3}\left(\omega_{2}\right) & ℑ\sigma_{4}\left(\omega_{2}\right)\\ ℜ\sigma_{1}\left(\omega_{3}\right) & ℑ\sigma_{2}\left(\omega_{3}\right) & ℜ\sigma_{3}\left(\omega_{3}\right) & ℑ\sigma_{4}\left(\omega_{3}\right)\\ ℜ\sigma_{1}\left(\omega_{4}\right) & ℑ\sigma_{2}\left(\omega_{4}\right) & ℜ\sigma_{3}\left(\omega_{4}\right) & ℑ\sigma_{4}\left(\omega_{4}\right) \end{array}\right]\\ & = & \sqrt{2}\left[\begin{array}{cccc} ℜ(1-\zeta_{5}) & ℑ(1-\zeta_{5}^{4}) & ℜ(1-\zeta_{5}^{2}) & ℑ(1-\zeta_{5}^{3})\\ ℜ(\zeta_{5}-\zeta_{5}^{2}) & ℑ(\zeta_{5}^{4}-\zeta_{5}^{3}) & ℜ(\zeta_{5}^{2}-\zeta_{5}^{4}) & ℑ(\zeta_{5}^{3}-\zeta_{5})\\ ℜ(\zeta_{5}^{2}-\zeta_{5}^{3}) & ℑ(\zeta_{5}^{3}-\zeta_{5}^{2}) & ℜ(\zeta_{5}^{4}-\zeta_{5}) & ℑ(\zeta_{5}-\zeta_{5}^{4})\\ ℜ(\zeta_{5}^{3}-\zeta_{5}^{4}) & ℑ(\zeta_{5}^{2}-\zeta_{5}) & ℜ(\zeta_{5}-\zeta_{5}^{3}) & ℑ(\zeta_{5}^{4}-\zeta_{5}^{2}) \end{array}\right]. \end{array}$$

*The matrix $D_{\alpha }$ is the diagonal matrix $\mathrm{diag}\left(\sqrt{\sigma_{1}\left(\alpha \right)},\sqrt{\sigma_{2}\left(\alpha \right)},\sqrt{\sigma_{3}\left(\alpha \right)},\sqrt{\sigma_{4}\left(\alpha \right)}\right)$. In order to show that $\left(\rho^{-1}\left(C\right),b_{\alpha }\right)$ is integral, we compute the Gram matrix which is proposed in (47). Define $v={\left(v_{1},v_{2},v_{3},v_{4}\right)}^{t}={\left(1,\zeta_{5},\zeta_{5}^{2},\zeta_{5}^{3}\right)}^{t}$ and $\omega ={\left(\omega_{1},\omega_{2},\omega_{3},\omega_{4}\right)}^{t}=\left(1-\zeta_{5}\right)v$, then*

$$G_{C}=\left[\begin{array}{cc} {\mathrm{Tr}}_{K/Q}\left(5\alpha vv^{†}\right) & {\mathrm{Tr}}_{K/Q}\left(2\alpha v\omega^{†}\right)\\ {\mathrm{Tr}}_{K/Q}\left(2\alpha \bar{\omega }v^{t}\right) & {\mathrm{Tr}}_{K/Q}\left(\alpha \omega \omega^{†}\right) \end{array}\right],$$

*in which*

(66) $$vv^{†}=\left[\begin{array}{c} 1\\ \zeta_{5}\\ \zeta_{5}^{2}\\ \zeta_{5}^{3} \end{array}\right]\left[\begin{array}{cccc} 1 & \zeta_{5}^{4} & \zeta_{5}^{3} & \zeta_{5}^{2} \end{array}\right]=\left[\begin{array}{cccc} 1 & \zeta_{5}^{4} & \zeta_{5}^{3} & \zeta_{5}^{2}\\ \zeta_{5} & 1 & \zeta_{5}^{4} & \zeta_{5}^{3}\\ \zeta_{5}^{2} & \zeta_{5} & 1 & \zeta_{5}^{4}\\ \zeta_{5}^{3} & \zeta_{5}^{2} & \zeta_{5} & 1 \end{array}\right],$$

(67) $$v\omega^{†}=v\left(\bar{1-\zeta_{5}}\right)v^{†}=\left(1-\zeta_{5}^{4}\right)vv^{†},$$

(68) $$\bar{\omega }v^{t}=\left(\bar{1-\zeta_{5}}\right)\bar{v}v^{t}=\left(1-\zeta_{5}^{4}\right){\left(vv^{†}\right)}^{t},$$

(69) $$\omega \omega^{†}=\left(1-\zeta_{5}\right)\left(1-\zeta_{5}^{4}\right)\left(vv^{†}\right)=\left(3+\zeta_{5}^{2}+\zeta_{5}^{3}\right)vv^{†}.$$

*Using the additive property of the trace function, it is enough to find ${\mathrm{Tr}}_{K/Q}\left(\sqrt{5}\zeta_{5}^{i}\right)$, for i=0,1,…,4. We have*

$$\begin{array}{ccc} {\mathrm{Tr}}_{K/Q}\left(\sqrt{5}\right) & = & {\mathrm{Tr}}_{K/Q}\left(-1-2\zeta_{5}^{2}-2\zeta_{5}^{3}\right)=4\left(-1\right)+2+2=0,\\ {\mathrm{Tr}}_{K/Q}\left(\sqrt{5}\zeta_{5}\right) & = & {\mathrm{Tr}}_{K/Q}\left(2+\zeta_{5}+2\zeta_{5}^{2}\right)=8-1-2=5,\\ {\mathrm{Tr}}_{K/Q}\left(\sqrt{5}\zeta_{5}^{2}\right) & = & {\mathrm{Tr}}_{K/Q}\left(2\zeta_{5}+\zeta_{5}^{2}+2\zeta_{5}^{3}\right)=-2-1-2=-5,\\ {\mathrm{Tr}}_{K/Q}\left(\sqrt{5}\zeta_{5}^{3}\right) & = & {\mathrm{Tr}}_{K/Q}\left(-2-2\zeta_{5}-\zeta_{5}^{3}\right)=-8+2+1=-5,\\ {\mathrm{Tr}}_{K/Q}\left(\sqrt{5}\zeta_{5}^{4}\right) & = & {\mathrm{Tr}}_{K/Q}\left(1-\zeta_{5}-\zeta_{5}^{2}+\zeta_{5}^{3}\right)=4+1+1-1=5. \end{array}$$

*For example, we compute the upper left component of $G_{C}$ as follows:*

$$\begin{array}{ccc} {\mathrm{Tr}}_{K/Q}\left(5\alpha vv^{†}\right) & = & \left[\begin{array}{cccc} {\mathrm{Tr}}_{K/Q}\left(\sqrt{5}\right) & {\mathrm{Tr}}_{K/Q}\left(\sqrt{5}\zeta_{5}^{4}\right) & {\mathrm{Tr}}_{K/Q}\left(\sqrt{5}\zeta_{5}^{3}\right) & {\mathrm{Tr}}_{K/Q}\left(\sqrt{5}\zeta_{5}^{2}\right)\\ {\mathrm{Tr}}_{K/Q}\left(\sqrt{5}\zeta_{5}\right) & {\mathrm{Tr}}_{K/Q}\left(\sqrt{5}\right) & {\mathrm{Tr}}_{K/Q}\left(\sqrt{5}\zeta_{5}^{4}\right) & {\mathrm{Tr}}_{K/Q}\left(\sqrt{5}\zeta_{5}^{3}\right)\\ {\mathrm{Tr}}_{K/Q}\left(\sqrt{5}\zeta_{5}^{2}\right) & {\mathrm{Tr}}_{K/Q}\left(\sqrt{5}\zeta_{5}\right) & {\mathrm{Tr}}_{K/Q}\left(\sqrt{5}\right) & {\mathrm{Tr}}_{K/Q}\left(\sqrt{5}\zeta_{5}^{4}\right)\\ {\mathrm{Tr}}_{K/Q}\left(\sqrt{5}\zeta_{5}^{3}\right) & {\mathrm{Tr}}_{K/Q}\left(\sqrt{5}\zeta_{5}^{2}\right) & {\mathrm{Tr}}_{K/Q}\left(\sqrt{5}\zeta_{5}\right) & {\mathrm{Tr}}_{K/Q}\left(\sqrt{5}\right) \end{array}\right]\\ & = & \left[\begin{array}{cccc} 0 & 5 & -5 & -5\\ 5 & 0 & 5 & -5\\ -5 & 5 & 0 & 5\\ -5 & -5 & 5 & 0 \end{array}\right]. \end{array}$$

*Other components can be computed similarly and we have*

(70) $$G_{C}=\left[\begin{array}{cccccccc} 0 & 5 & -5 & -5 & -2 & 4 & 0 & -4\\ 5 & 0 & 5 & -5 & 2 & -2 & 4 & 0\\ -5 & 5 & 0 & 5 & -4 & 2 & -2 & 4\\ -5 & -5 & 5 & 0 & 0 & -4 & 2 & -2\\ -2 & 2 & -4 & 0 & -2 & 3 & -2 & -2\\ 4 & -2 & 2 & -4 & 3 & -2 & 3 & -2\\ 0 & 4 & -2 & 2 & -2 & 3 & -2 & 3\\ -4 & 0 & 4 & -2 & -2 & -2 & 3 & -2 \end{array}\right].$$

*Thus, $\left(\rho^{-1}\left(C\right),b_{\alpha }\right)$ is an integral lattice and it can be checked that $\det\left(G_{C}\right)=5^{4}$, that is a necessary condition for being 5-modular.*

### 6.2. Non-Existence of Modular Lattices from Prime-Power Cyclotomic Fields via Construction A

In this section, we review the construction of modular lattices in [80] using the provided algebraic tools in the previous sections. We consider $K=Q(\zeta_{n})$ and its maximal totally real subfield $K^{+}$, when *n* is a prime power or when *n* is composite. There are two approaches to construct modular lattices using algebraic number fields:

- Construction using ideal lattices [79,85,86,87],
- Construction using generalized Construction A, which was introduced in Section 4.2.

An ideal lattice is an ideal of a number field *K* together with a bilinear form satisfying an invariance relation [86]. Using ideal lattices in order to construct modular lattices has been investigated in [79,85,87]. Here, we concentrate on generalizing the results of Propositions 8 and 9 to obtain *d*-modular lattices using cyclotomic number fields. As far as we are aware, the generalizations of the above results to $K=Q(\zeta_{p^{r}})$ and $K^{+}=Q\left(\zeta_{p^{r}}+\zeta_{p^{r}}^{-1}\right)$, with r>1, or generalization to the cases that $K=Q(\zeta_{n})$, with $n\neq p^{r}$ for a prime number *p*, have not been addressed in the literature. In the following theorems, we consider all of these cases.

**Theorem** **14.** *Let $K=Q(\zeta_{p^{r}})$, with r>1 and p an odd prime number, be the cyclotomic field with the ring of integers $O_{K}=Z\left[\zeta_{p^{r}}\right]$. We have that K is a CM field and the prime p totally ramifies in K as $pO_{K}=P^{p^{r-1}\left(p-1\right)}$, with residue field $O_{K}/P≅F_{p}$, where $P=(1-\zeta_{p^{r}})$. Let $C\subset F_{p}^{N}$ be an (N,k) self-dual code over $F_{p}$. Then, $\left(\rho^{-1}\left(C\right),b_{1/p}\right)$ with $b_{1/p}\left(x,y\right)=\sum_{i=1}^{N}{\mathrm{Tr}}_{K/Q}\left(x_{i}{\bar{y}}_{i}/p\right)$, is d-modular if and only if d=1 and r=1.*

**Proof.**  See Appendix A.3. □

**Theorem** **15.** *Let $K^{+}=Q\left(\zeta_{p^{r}}+\zeta_{p^{r}}^{-1}\right)$, with r>1 and p an odd prime number, be the totally real maximal subfield of a cyclotomic field with the ring of integers $O_{K^{+}}=Z\left[\zeta_{p^{r}}+\zeta_{p^{r}}^{-1}\right]$. We have that $K^{+}$ is a totally real number field and the prime p totally ramifies in $K^{+}$ as $pO_{K^{+}}=p^{\frac{p^{r-1}\left(p-1\right)}{2}}$, with residue field $O_{K^{+}}/p≅F_{p}$, where $p=(2-\zeta_{p^{r}}-\zeta_{p^{r}}^{-1})$. Let $C\subset F_{p}^{N}$ be an (N,k) self-dual code over $F_{p}$. Then, $\left(\rho^{-1}\left(C\right),b_{1/p}\right)$ with $b_{1/p}\left(x,y\right)=\sum_{i=1}^{N}{\mathrm{Tr}}_{K/Q}\left(x_{i}y_{i}/p\right)$ is d-modular if and only if d=1 and r=1.*

**Proof.**  See Appendix A.4 □

In other cases, that is when $K=Q(\zeta_{n})$ and $n\neq p^{r}$ for an odd prime number *p*, making a general decision is not easy. In the sequel, we present an example that indicates the complexity of this general case.

**Example** **4.** *Let $K=Q(\zeta_{6})$ with the ring of integers $O_{K}=Z\left[\zeta_{6}\right]$. The minimal polynomial of $\zeta_{6}$ is $\Phi_{6}\left(X\right)=X^{2}-X+1$ and using Proposition 2, we conclude that 2 is inert in $O_{K}$; that is, $P=2O_{K}$ is a prime ideal of $O_{K}$ with residue field $O_{K}/P≅F_{4}$. Define $\rho :O_{K}^{N}\to F_{4}^{N}$, as the componentwise reduction modulo P and consider the 2N-dimensional lattice $\Gamma_{C}=\left(\rho^{-1}\left(C\right),b_{1/2}\right)$, where $b_{1/2}\left(x,y\right)=\sum_{i=1}^{N}{\mathrm{Tr}}_{K/Q}\left(x_{i}y_{i}/2\right)$ and C is an (N,k) linear code over $F_{4}$. The volume of $\Gamma_{C}$ is $\mathrm{vol}\left(\Gamma_{C}\right)={\left|d_{K}\right|}^{\frac{N}{2}}4^{N-k}2^{-N}$, where $d_{K}$ is given in (25)*

(71) $$d_{K}={\left(-1\right)}^{\phi (6)/2}\frac{6^{\phi (6)}}{\prod_{p|6}p^{\phi (6)/(p-1)}}=\frac{-6^{2}}{2^{2}3^{1}}=-3.$$

Thus, $vol\left(\Gamma_{C}\right)=3^{\frac{N}{2}}2^{N-2k}$. Equating $vol(\frac{1}{\sqrt{d}}\Gamma_{C})$ and $vol(\Gamma_{C}^{*})$ gives us $d^{N}=3^{N}4^{N-2k}$. If we consider a self dual code C over $F_{4}$, k=N/2 and d=3. In principal, it seems that construction of a family of 3-modular lattices is possible. However, this is not true and we show this providing a counter example. Consider the self-dual code $C=\langle (1,\omega ,\omega +1,0),(1,1,1,1)\rangle$ [52], where ω is the primitive element of $F_{4}$ and $\omega^{2}+\omega +1=0$. The Galois group of K/Q is $G=\{\sigma_{1},\sigma_{2}\}$, where $\sigma_{1}$ is the identity map and $\sigma_{2}$ is complex conjugation. The set $\left\{v_{1}=1,v_{2}=\zeta_{6}\right\}$ forms a Z-basis for $O_{K}$ and $\left\{\omega_{1}=2,\omega_{2}=2\zeta_{6}\right\}$ forms a Z-basis for P. The map $\rho :O_{K}^{N}\to F_{4}^{N}$ sends $\zeta_{6}$ to ω and using the following generator matrix for C

(72) $$\left[\begin{array}{cc} I_{2} & A\;\;(\mathrm{mod}\;P) \end{array}\right]=\left[\begin{array}{cccc} 1 & 0 & \omega & \omega +1\\ 0 & 1 & \omega +1 & \omega \end{array}\right],$$

*we obtain*

(73) $$A=\left[\begin{array}{cc} \zeta_{6} & \zeta_{6}+1\\ \zeta_{6}+1 & \zeta_{6} \end{array}\right].$$

*Using the above matrix A and the matrices in Proposition 5, gives us the generator matrix of $\Gamma_{C}$*

(74) $$M=\sqrt{2}\left[\begin{array}{cc} ℜ\sigma_{1}\left(1\right) & ℑ\sigma_{2}\left(1\right)\\ ℜ\sigma_{1}\left(\zeta_{6}\right) & ℑ\sigma_{2}\left(\zeta_{6}\right) \end{array}\right]=\sqrt{2}\left[\begin{array}{cc} 1 & 0\\ \frac{1}{2} & \frac{-\sqrt{3}}{2} \end{array}\right],$$

(75) $$M_{p}=\sqrt{2}\left[\begin{array}{cc} ℜ\sigma_{1}\left(2\right) & ℑ\sigma_{2}\left(2\right)\\ ℜ\sigma_{1}\left(2\zeta_{6}\right) & ℑ\sigma_{2}\left(2\zeta_{6}\right) \end{array}\right]=\sqrt{2}\left[\begin{array}{cc} 2 & 0\\ 1 & -\sqrt{3} \end{array}\right],$$

(76) $$\begin{array}{ccc} A\tilde{\tilde{⊗}}M & = & \sqrt{2}\left[\begin{array}{cccc} ℜ\sigma_{1}\left(A_{1}⊗v\right) & ℑ\sigma_{2}\left(A_{1}⊗v\right) & ℜ\sigma_{1}\left(A_{2}⊗v\right) & ℑ\sigma_{2}\left(A_{2}⊗v\right) \end{array}\right]\\ & = & \sqrt{2}\left[\begin{array}{cccc} \frac{1}{2} & \frac{-\sqrt{3}}{2} & \frac{3}{2} & \frac{-\sqrt{3}}{2}\\ \frac{-1}{2} & \frac{-\sqrt{3}}{2} & 0 & -\sqrt{3}\\ \frac{3}{2} & \frac{-\sqrt{3}}{2} & \frac{1}{2} & \frac{-\sqrt{3}}{2}\\ 0 & -\sqrt{3} & \frac{-1}{2} & \frac{-\sqrt{3}}{2} \end{array}\right], \end{array}$$

(77) $$D_{\alpha }=\left[\begin{array}{cc} \sqrt{\sigma_{1}\left(\frac{1}{2}\right)} & 0\\ 0 & \sqrt{\sigma_{2}\left(\frac{1}{2}\right)} \end{array}\right]=\frac{1}{\sqrt{2}}I_{2},$$

*where $\tilde{\tilde{⊗}}$ was defined in (45). Thus, the generator matrix of $\Gamma_{C}$ is*

(78) $$M_{C}=\left[\begin{array}{cccccccc} 1 & 0 & 0 & 0 & \frac{1}{2} & \frac{-\sqrt{3}}{2} & \frac{3}{2} & \frac{-\sqrt{3}}{2}\\ \frac{1}{2} & \frac{-\sqrt{3}}{2} & 0 & 0 & \frac{-1}{2} & \frac{-\sqrt{3}}{2} & 0 & -\sqrt{3}\\ 0 & 0 & 1 & 0 & \frac{3}{2} & \frac{-\sqrt{3}}{2} & \frac{1}{2} & \frac{-\sqrt{3}}{2}\\ 0 & 0 & \frac{1}{2} & \frac{-\sqrt{3}}{2} & 0 & -\sqrt{3} & \frac{-1}{2} & \frac{-\sqrt{3}}{2}\\ 0 & 0 & 0 & 0 & 2 & 0 & 0 & 0\\ 0 & 0 & 0 & 0 & 1 & -\sqrt{3} & 0 & 0\\ 0 & 0 & 0 & 0 & 0 & 0 & 2 & 0\\ 0 & 0 & 0 & 0 & 0 & 0 & 1 & -\sqrt{3} \end{array}\right].$$

*Computing the Gram matrix $G_{C}=M_{C}M_{C}^{t}$ indicates that $\Gamma_{C}$ is not integral and consequently is not modular.*

The only case that was not considered in the previous theorems is the case where $n=2^{r}$, for r>1. In this case, we were unsuccessful in changing *r* and α in order to obtain a family of *d*-modular lattices. We should point out that when considering applications in information security only special values of *d* are accepted, more precisely, d=1,2,3,5,6,7,11,14,15 and 23 [53]. We could not find any modular lattice in our trials using cyclotomic number fields (with non-prime orders) and Construction A, that fulfil these conditions. Thus, these remain open problems.

**Example** **5.** *Let $K=Q\left(\zeta_{4}\right)=Q\left(i\right)$ be the cyclotomic field of order* 4 *with the ring of integers $O_{K}=Z\left[i\right]$. Then, K is a CM field and the prime p=5 splits completely in K as $5O_{K}=P_{1}P_{2}$, with residue field $O_{K}/P_{i}≅F_{5}$, for i=1,2, where $P_{1}=5O_{K}+\left(i-2\right)O_{K}$ and $P_{2}=5O_{K}+\left(i-3\right)O_{K}$. Let $C\subset F_{5}^{N}$ be an (N,k) self-dual code over $F_{5}$. Then, $\left(\rho^{-1}\left(C\right),b_{1/2}\right)$ with $b_{1/2}\left(x,y\right)=\sum_{i=1}^{N}{\mathrm{Tr}}_{K/Q}\left(x_{i}{\bar{y}}_{i}/2\right)$ is an integral lattice and its determinant is $5^{N}$. Thus, $\rho^{-1}\left(C\right)$ has some necessary conditions to be a 5-modular lattice. However, one can check that this lattice is not 5-modular.*

### 6.3. Secrecy Performance Characterization via Secrecy Gain and Flatness Factor

Computing the secrecy gain for unimodular lattices has been considered in [40]. Then, in [30,52], the authors propose some methods, based on the techniques introduced in [57] for calculating the theta series of modular lattices, to calculate the weak secrecy gain of 2,3-modular lattices and 5-modular lattices, respectively. The introduced approaches to obtain a closed form expression for the theta series of modular lattices can be divided into two different cases: the modular form approach and the weight enumerator approach. The modular form approach relies on the fact that the theta series of an *ℓ*-modular lattice belongs to the space of modular forms generated by some basic functions, which gives a decomposition formula. The formula is given for *ℓ*-modular (possibly odd) lattices, which holds for the specific values of ℓ=1,2,3,5,6,7,11,14,15,23 [30,57]. A weight enumerator approach exploits the connection between the weight enumerator of a self-dual code and the theta series of a lattice constructed from this code. The theta series of a 5-modular lattice Γ, with dimension n=2t, can be written as [30,57], Lemma 2

(79) $$\Theta_{\Gamma }\left(\tau \right)=f_{1}{\left(\tau \right)}^{t}\sum_{i=0}^{\left\lfloor \frac{5t}{8}\right\rfloor }a_{i}f_{2}{\left(\tau \right)}^{i},$$

where $f_{1}\left(\tau \right)=\vartheta_{3}\left(\tau \right)\vartheta \left(5\tau \right)$ and

(80) $$f_{2}\left(\tau \right)={\left(\frac{\eta \left(\frac{\tau }{2}\right)\eta \left(\frac{5\tau }{2}\right)\eta \left(2\tau \right)\eta \left(10\tau \right)}{\eta {\left(\tau \right)}^{2}\eta {\left(5\tau \right)}^{2}}\right)}^{\frac{8}{2}},$$

in which η is the Dedekind eta function which is defined by [88], Chapter 3

(81) $$\eta \left(\tau \right)=q^{\frac{1}{12}}\prod_{m=1}^{\infty }\left(1-q^{2m}\right).$$

Equation (79) gives the theta series in terms of the unknown values $a_{i}$’s. An interesting computational approach has been proposed in [30] for the cases that ℓ=2,3. In their approach, the Gram matrix has been computed and inputted it to Magma [89] to generate the lattice Λ. The first few terms of $\Theta_{\Lambda }\left(\tau \right)$ have been obtained (using the command ThetaSeries(Λ,0,Ord);). Then, by solving a linear system of equations in terms of the unknowns $a_{i}$’s, the theta series has been obtained as polynomials in terms of basic functions $\vartheta_{1},\vartheta_{2},\vartheta_{3}$ which are implemented in Mathematica [90]. Then, the weak secrecy gains have been approximated using Mathematica. We are not able to use this approach for the obtained lattice in Example 3, because the Gram matrix of our lattices are not positive definite and it is a necessary condition for Magma to compute the theta series; we are not aware of other computer algebra packages for this task.

The main conclusion about the connection between the weak secrecy gain of the lattice and other lattice parameters has been reported in [53] after studying many examples. This conclusion is summarized as follows [53], Remark 4.12:

1. When the dimension increases, the weak secrecy gain $\chi_{\Lambda }$ tends to increase, which has been proven for unimodular lattices [40].
2. Fixing dimension and level *d*, a large length for the shortest non-zero vector is more likely to induce a large $\chi_{\Lambda }$.
3. Fixing dimension, level *d* and the length of the shortest non-zero vector, a smaller kissing number gives a larger $\chi_{\Lambda }$. It was shown for unimodular lattices [40] that when the dimension *n* is fixed, n≤23, the secrecy gain is totally determined by the kissing number, and the lattice with the best secrecy gain is the one with the smallest kissing number.
4. Fixing dimension, the length of the shortest non-zero vector, kissing number, and a smaller level *d* gives a bigger $\chi_{\Lambda }$. However, the lattices with high level *d* are more likely to have a large length for the shortest non-zero vector.

### 6.4. Motivation for Indefinite Theta Series in Physical Layer Security

The construction of *p*-modular lattices in our framework inevitably produces lattices whose Gram matrices are not positive definite and have indefinite signature. While this is a natural outcome from the algebraic constraints imposed by the construction, it creates a fundamental obstacle: the classical theta series $\Theta_{\Lambda }\left(\tau \right)=\sum_{x\in \Lambda }q^{2Q(x)}$, $q=e^{\pi i\tau }$, is only absolutely convergent on the unit disk when the quadratic form *Q* is positive definite. For indefinite lattices, the summand does not decay fast enough along directions of positive norm, and the series diverges on the unit circle. This prevents the direct use of standard tools from modular lattice theory for computing secrecy gain, flatness factor, or any performance metric that depends on the analytic behavior of $\Theta_{\Lambda }\left(\tau \right)$.

Mathematical advances in the theory of indefinite theta series, most notably the work of Zwegers [56] and the general framework developed in [55], provide a way around this difficulty. Instead of attempting to force convergence by restricting the lattice or modifying the construction, one replaces the classical theta series by a *modular completion* built from special kernels that regularize the contribution of directions with positive norm. These kernels are expressed in terms of generalized error functions, such as $E_{1}$, $M_{1}$ in the Lorentzian case and their higher-dimensional analogues $E_{2}$, $M_{2}$ for signature (2,n−2) lattices. The key insight is that these functions interpolate smoothly between the discontinuous sign functions that appear in naive truncations of the lattice sum, while still satisfying Vignéras’ differential equation and thus preserving modular covariance.

From the perspective of physical-layer security, this development is highly relevant. The secrecy rate and the eavesdropper’s decoding probability are governed by the *flatness factor* of the lattice, which in turn depends on the behavior of its theta series near the unit circle. As we discussed earlier, for positive definite lattices, the flatness factor admits a clean expression in terms of $\Theta_{\Lambda }\left(\tau \right)$ evaluated at purely imaginary arguments. However, for the indefinite lattices produced by our construction, the classical theta series is not defined in this regime. The modularly completed indefinite theta series, on the other hand, *is* well defined and exhibits controlled analytic behavior even when the underlying quadratic form has mixed signature.

This observation motivates the study of indefinite theta series as a natural analytic tool for characterizing secrecy performance. The completed theta series captures the oscillatory structure of the lattice while regularizing divergent contributions, allowing us to define meaningful analogues of the flatness factor and secrecy gain. Moreover, the modular properties of the completed series provide structural constraints that can be exploited to bound or approximate the eavesdropper’s error probability. In particular, the smoothing induced by the generalized error functions plays a role analogous to artificial noise in physical-layer security: it suppresses the contribution of directions that would otherwise leak information to the eavesdropper.

In summary, although our construction yields indefinite lattices for which the classical theta series is not applicable, the modern theory of indefinite theta series offers a principled replacement. This framework not only restores analytic control but also reveals deeper connections between lattice geometry, modularity, and secrecy performance. Consequently, the study of indefinite theta series is not merely a mathematical necessity but a promising avenue for developing new secrecy metrics and designing lattice codes optimized for physical layer security.

#### Indefinite Quadratic Forms, Vignéras’ Operator, and Generalized Error Functions

The analytic framework for handling theta series associated with indefinite lattices relies on several structural definitions. These notions originate in the modern theory of indefinite theta series and generalized error functions, and they provide the mathematical backbone for constructing modularly well-behaved completions of divergent lattice sums [55].

Let Λ be a lattice equipped with a symmetric bilinear form B(·,·) and associated quadratic form Q(x)=B(x,x), where x∈Λ⊗R. The form *Q* is said to have *signature*$\left(n_{+},n_{-}\right)$ if it has $n_{+}$ positive and $n_{-}$ negative eigenvalues. In our construction of *p*-modular lattices, the Gram matrices naturally acquire mixed signature, which places us in the regime $n_{+}\geq 1$ where classical theta series fail to converge.

**Definition** **17** (Vignéras’ Operator)**.** *Let* Λ * be a lattice equipped with a symmetric bilinear form B(·,·) of signature $\left(n_{+},n_{-}\right)$, and let Q(x)=B(x,x) denote the associated quadratic form. We write $B^{-1}$ for the bilinear form on the dual space whose matrix is the inverse of the matrix of B. Following the general framework of Vignéras, we introduce the second–order differential operator [54]*

(82) $$W_{\lambda }\Phi \left(x\right)\;:=\;\Delta_{B^{-1}}\Phi \left(x\right)\;+\;2\pi \;x\cdot \nabla_{x}\Phi \left(x\right)\;-\;2\pi \lambda \;\Phi \left(x\right),$$

*where $\Delta_{B^{-1}}$ is the Laplacian associated with $B^{-1}$ and $x\cdot \nabla_{x}$ is the Euler operator. A function Φ(x) is said to be a Vignéras kernel of weight λ if it satisfies the differential equation*

(83) $$W_{\lambda }\Phi \left(x\right)=0.$$

Solutions of (83) play a central role in the construction of modular completions of indefinite theta series. In particular, when Φ satisfies suitable decay conditions, the associated theta series transforms as a Jacobi form of weight $\lambda +\frac{n}{2}$, even when the underlying quadratic form *Q* has mixed signature.

**Definition** **18** (Indefinite Theta Series with Kernel [55])**.** *Given a kernel Φ(x) satisfying mild growth conditions, one defines the regularized theta series*

(84) $$\begin{array}{c} \Theta_{\Phi }\left(\tau ,b,c\right)=\sum_{k\in \Lambda +P/2}{\left(-1\right)}^{B(k,P)}\;q^{-2Q(k+b)}\;e^{2\pi i\;B(c,k+b)}\;\Phi \left(k+b\right), \end{array}$$

*where P is a characteristic vector and (b,c) are real shifts.*

When Φ is locally constant (e.g., a sign function), the series is holomorphic but not modular; when Φ solves Vignéras’ equation, the series is modular but non-holomorphic. The modular completion replaces the discontinuous kernel by a smooth one.

**Definition** **19** (Generalized Error Functions [55])**.** *For signature (1,n−1), the classical error function*

(85) $$\begin{array}{c} E_{1}\left(u\right)=\mathrm{Erf}\left(\sqrt{\pi }\;u\right), \end{array}$$

*where $\mathrm{Erf}\left(x\right)=\frac{2}{\sqrt{\pi }}\int_{0}^{x}e^{-t^{2}}\;dt$, and its complementary counterpart*

(86) $$\begin{array}{c} M_{1}\left(u\right)=-\mathrm{sign}\left(u\right)\;\mathrm{Erfc}(|u|\sqrt{\pi }), \end{array}$$

*in which $\mathrm{Erfc}\left(x\right)=1-\mathrm{Erf}\left(x\right)=\frac{2}{\sqrt{\pi }}\int_{x}^{\infty }e^{-t^{2}}\;dt$, provide the smoothing needed to regularize the theta series.*

In higher signature, one introduces *double error functions* $E_{2}$ and $M_{2}$, depending on two real variables $\left(u_{1},u_{2}\right)$ and a geometric parameter controlling the angle between the associated hyperplanes. These functions interpolate between piecewise-constant sign kernels and smooth solutions of Vignéras’ equation on $R^{2}$.

**Definition** **20** (Modular Completion [55])**.** *Given a discontinuous kernel $\Phi_{\mathrm{disc}}$ (typically built from sign functions that restrict the lattice to a cone), its modular completion is constructed by replacing each sign term with the corresponding smooth error function:*

(87) $$\begin{array}{c} \Phi_{\mathrm{comp}}\left(x\right)=\Phi_{\mathrm{disc}}\left(x\right)+(correction\;terms\;built\;from\;M_{1},\;M_{2},\;\mathrm{etc}.). \end{array}$$

The resulting completed theta series is modular and well-behaved on the unit circle, even when *Q* is indefinite. These definitions allow us to reinterpret the divergent theta series associated with our *p*-modular constructions as *regularized indefinite theta series*. This provides a mathematically rigorous pathway to define secrecy-relevant quantities—such as flatness factor and secrecy gain—even when the underlying lattice is not positive definite.

### 6.5. Quantitative Comparison of Lattice Families for PLS

To position the proposed *p*-modular lattices within the broader framework of algebraic lattice design for physical-layer security, we compare their principal structural invariants like signature, discriminant, and modularity constant, which together with the minimum norm and kissing number, strongly influence shaping behavior, coding efficiency, and the analytic feasibility of secrecy metrics. Table 2 summarizes these invariants across classical and newly introduced families. Beyond these structural aspects, secrecy-relevant analytic behavior must be interpreted in light of the dependencies identified in [53], Remark 4.12: the weak secrecy gain $\chi_{\Lambda }$ tends to increase with dimension; it improves with larger minimum norm; it decreases with larger kissing number when dimension, level, and minimum norm are fixed; and, for fixed geometric parameters, smaller level *d* generally yields larger $\chi_{\Lambda }$, while higher levels often enable larger minimum norms. These principles provide a quantitative framework for evaluating the proposed *p*-modular lattices.

For $K=Q(\zeta_{p})$, the discriminant satisfies $\left|\mathrm{disc}\left(K\right)\right|=p^{p-2}$, and the modularity constant *p* imposes strong arithmetic constraints on the admissible combinations of volume, minimum norm, and kissing number. Moreover, the total ramification of *p* in $Q(\zeta_{p})$ facilitates Construction A lattices for which the minimum norm can be improved through the underlying code while keeping the growth of the kissing number analytically tractable. In this sense, the *p*-modular family occupies a structurally advantageous region of the secrecy-design landscape: the dimension grows linearly with *p*, the minimum norm is tunable via the code, and the kissing number can be studied and bounded through the explicit cyclotomic embedding.

Indeed, in the cyclotomic setting, the kissing number of the associated lattices can be analyzed with finer arithmetic control by exploiting the explicit Q-embedding of $O_{K}$ into Euclidean space. For $K=Q(\zeta_{p})$, the canonical embedding $\sigma :K↪C^{(p-1)/2}$ (or its realification into $R^{p-1}$) is determined by the embeddings $\sigma_{j}\left(\zeta_{p}\right)=\exp\;\left(\frac{2\pi ij}{p}\right)$, $1\leq j\leq \frac{p-1}{2}$, where each $\sigma_{j}$ is paired with its complex conjugate. Writing $x\in O_{K}$ in the power basis $x=\sum_{k=0}^{p-2}a_{k}\zeta_{p}^{k}$ with $a_{k}\in Z$, the Euclidean norm of its image is

(88) $$\begin{array}{c} {∥\sigma \left(x\right)∥}^{2}=\sum_{j=1}^{(p-1)/2}{\left|\sigma_{j}\left(x\right)\right|}^{2}=\sum_{j=1}^{(p-1)/2}{\left|\sum_{k=0}^{p-2}a_{k}\exp\;\left(\frac{2\pi ijk}{p}\right)\right|}^{2}, \end{array}$$

which is an explicit positive-definite quadratic form in the integer vector $\left(a_{0},\ldots ,a_{p-2}\right)$. The Construction A lattices considered in this work are obtained by embedding codewords with coordinates in (the image of) $O_{K}$ and then scaling by a suitable power of the prime above *p*, so any non-zero lattice vector corresponds to a tuple $\left(x_{1},\ldots ,x_{N}\right)\in O_{K}^{N}$ whose Euclidean norm is given by $\sum_{i=1}^{N}{∥\sigma \left(x_{i}\right)∥}^{2}$, again an explicit quadratic form in the underlying integer coefficients. The minimum norm $\lambda_{1}\left(\Lambda \right)$ is therefore the minimum of this quadratic form over a discrete subset of $Z^{(p-1)N}$ determined by the code constraints, and candidate shortest vectors can be characterized by bounding the integer coefficients $a_{k}$.

From the arithmetic side, the algebraic norm $N_{K/Q}\left(x\right)=\prod_{j=1}^{p-1}\sigma_{j}\left(x\right)$ is an integer and satisfies an inequality of the form

(89) $$\begin{array}{c} |N_{K/Q}\left(x\right)|\leq C_{p}\;{∥\sigma \left(x\right)∥}^{p-1}, \end{array}$$

for a constant $C_{p}$ depending only on *p*. Thus, any bound on the Euclidean norm imposes a corresponding bound on $|N_{K/Q}\left(x\right)|$. Consequently, the set of $x\in O_{K}$ that can contribute to shortest vectors lies in a finite, explicitly describable subset of $O_{K}$ cut out by simultaneous constraints on ${∥\sigma \left(x\right)∥}^{2}$ and $|N_{K/Q}\left(x\right)|$. Moreover, the Galois group $\mathrm{Gal}\left(K/Q\right)≅{\left(Z/pZ\right)}^{\times }$ acts transitively on the embeddings $\sigma_{j}$ and preserves the Euclidean norm, so (under the natural assumption that the lattice and the code constraints are stable under this action) if *x* yields a shortest vector, then so do all its Galois conjugates, which form orbits whose sizes divide p−1. As a consequence, the multiplicity of shortest vectors (and hence the kissing number) can be expressed in terms of the number of Galois orbits of algebraic integers (or code-constrained tuples) achieving $\lambda_{1}\left(\Lambda \right)$, each orbit contributing a controlled number of vectors. Altogether, the explicit quadratic form arising from the cyclotomic embedding, the finiteness imposed by algebraic norm bounds, and the orbit structure induced by the Galois action ensure that both the minimum norm and the kissing number of the resulting *p*-modular lattices can be studied and bounded through the cyclotomic embedding, rather than treated as opaque geometric invariants of an arbitrary Euclidean lattice.

Although the analytic lift of these lattices has mixed signature, which makes classical positive-definite theta-series techniques inapplicable, recent advances on modular completions of indefinite theta series provide a natural analytic framework for assessing their secrecy performance. Taken together, these structural constraints and analytic tools indicate that the proposed *p*-modular lattices are well aligned with the geometric and arithmetic features empirically associated with large secrecy gain, and they therefore constitute a technically robust and theoretically well-motivated class of lattices for high-dimensional physical-layer security.

### 6.6. Open Problems and Future Research Directions

The interplay between indefinite theta series and physical-layer security opens a number of intriguing research avenues. A central open problem is the development of secrecy metrics, such as generalized flatness factors or secrecy gains, that remain analytically meaningful when the underlying lattice has mixed signature and the classical theta series diverges. Closely related is the challenge of characterizing the behavior of modular completions built from higher-dimensional error functions (e.g., $E_{2}$ and $M_{2}$) in channel regimes relevant to wiretap coding, particularly when the noise distribution interacts nontrivially with the time-like and space-like directions of the lattice. Another promising direction is the design of coding schemes whose secrecy performance can be directly optimized through the geometry of indefinite cones, potentially leveraging the structural constraints imposed by Vignéras’ equation. Extending these ideas to signatures $\left(n_{+},n_{-}\right)$ with $n_{+}>2$, where triple and higher-order generalized error functions arise, remains largely unexplored and may reveal new analytically tractable lattice families. Finally, bridging the gap between the abstract modular-analytic theory and practical wiretap coding through numerical approximations, simulation frameworks, or machine-assisted optimization of indefinite kernels represents a fertile direction for future research.

## 7. Discussion and Integration into Modern Wireless Systems

The evolution toward next-generation wireless networks is characterized by the adoption of advanced paradigms that fundamentally reshape wireless communication. Within this landscape, lattice-based PLS offers both significant opportunities and formidable challenges. Modern architectures such as multi-antenna systems (MIMO and massive MIMO), RIS, and ML–driven optimization are no longer peripheral enhancements but central pillars of wireless design. Their integration with algebraic lattice coding introduces new dimensions of secrecy: spatial degrees of freedom, programmable propagation environments, and data-driven adaptation can be harnessed to reinforce the confusion of eavesdroppers while sustaining reliability for legitimate users [91]. This section explores how the code-based wiretap approaches like lattice-code surveyed earlier can be adapted to, and enriched by, these emerging technologies. For each paradigm, we highlight recent advances, analyze how lattice coset structures interact with the new physical and algorithmic features, and identify open research directions that define the frontier of secure 6G system design.

### 7.1. MIMO and Massive MIMO Deployment

Massive MIMO has emerged as a cornerstone of 5G and 6G wireless systems, offering unprecedented spectral efficiency and reliability by exploiting large antenna arrays to serve multiple terminals simultaneously. Beyond throughput, these spatial degrees of freedom provide new opportunities for physical-layer security. By carefully designing precoders and injecting artificial noise (AN), transmitters can degrade the eavesdropper’s channel while maintaining high- quality links for legitimate users. Recent works have demonstrated that secrecy capacity in massive MIMO can be significantly improved by exploiting channel hardening and favorable propagation. For example, refs. [92,93] analyzed secrecy in spherical-wave channels and showed that near-field beamforming can be leveraged to enhance PLS in practical deployments. Similarly, ref. [94] studied secrecy guard zones in ultra-reliable low-latency communications (uRLLC), highlighting how dense antenna deployments can enforce spatial secrecy constraints under strict latency requirements.

While these approaches rely on spatial processing, lattice codes provide an algebraic complement. Nested lattice coset coding introduces structured randomness that confuses the eavesdropper independently of channel state information (CSI) assumptions. Semantically secure lattice codes for compound MIMO channels, as developed by [95], demonstrate that secrecy can be achieved even under partial or uncertain CSI. These constructions align naturally with massive MIMO, where imperfect CSI is common due to pilot contamination and feedback delays. By embedding cyclotomic p-modular lattices (with p≡1(mod4)) into space–time blocks, one can exploit unit-group rotations and Minkowski embeddings to maintain low flatness factor at Eve while preserving diversity and rate at the receiver. The dual lattice’s theta series governs Eve’s confusion, and when combined with AN in unused spatial dimensions, secrecy gain can be amplified.

Cell-free massive MIMO extends these ideas by distributing many access points (APs) to jointly serve users via coherent transmission. This architecture inherently reduces Eve’s ability to gain coherent combining benefits in passive scenarios. Ref. [96] showed that secure transmission in cell- free systems can be maintained against active eavesdroppers by coordinating AN and robust power control. More recently, ref. [97] analyzed secrecy rate degradation under pilot contamination and proposed secure pilot design strategies for cell-free networks. These findings suggest that lattice-coded pilots, constructed from collision-resistant projections of cyclotomic lattices, could further mitigate spoofing attacks, while coset-coded payloads ensure resilience against residual leakage. In ultra-dense cell-free deployments, ref. [96] demonstrated that secrecy can be preserved even under hardware impairments by adopting rate-splitting multiple access (RSMA), opening avenues for lattice-coded RSMA schemes.

From a lattice perspective, space–time lattice designs remain particularly attractive. Semantically secure lattice codes for compound MIMO channels [94] and design criteria for MIMO wiretap channels [98] provide theoretical foundations for integrating algebraic lattices into multi-antenna secrecy. By aligning legitimate codewords with the receiver’s dominant singular vectors and dispersing Eve’s projections through lattice rotations, one can systematically engineer flatness factors that remain low at Eve. In multi-receiver wiretap scenarios, refs. [99,100] characterized optimal encoding orders for Gaussian MIMO channels, offering guidance for scheduling lattice layers across streams to maintain confidentiality. These results highlight the synergy between algebraic lattice coding and massive MIMO: spatial degrees of freedom create controllable subspaces for secrecy-aware transmission, while structured cosets ensure persistent confusion at Eve independent of beamforming imperfections.

The trade-offs are clear. Multi-antenna lattice precoding can significantly improve secrecy capacity but requires careful CSI acquisition and high-complexity decoding. Sphere decoding scales poorly with dimension, and while massive MIMO provides many antennas, practical receivers often limit effective dimensionality per codeword to meet latency constraints. Hybrid designs, such as block-diagonal space–time lattices or multi-layer coset coding with short lattices, can deliver secrecy gains without prohibitive complexity. Emerging ML-aided lattice decoders may further reduce decoding burden, though interpretability and robustness against adversarial attacks remain open challenges. Ultimately, joint precoder–lattice co-design under imperfect CSI, pilot contamination, and energy constraints offers a realizable path to secrecy gains in 5G/6G MIMO systems.

### 7.2. RIS-Aided Communications

Reconfigurable intelligent surfaces change the secrecy game by turning the propagation environment into a controllable design variable. An RIS is a planar array of passive elements whose reflection coefficients (amplitude and/or phase) can be programmed to reshape multipath, steer energy, and create constructive or destructive interference patterns at chosen spatial locations. For physical-layer security, this capability enables a new class of defenses: instead of relying only on transmitter beamforming or artificial noise, the network can program the channel so that the legitimate receiver sees a strengthened, well-conditioned effective lattice channel while the Eve sees a scrambled, low-mutual-information projection. This programmable geometry is a natural partner for algebraic lattice codes, because lattice secrecy metrics (flatness factor, secrecy gain, and dual-lattice theta series) are fundamentally geometric and therefore directly affected by RIS-induced channel transformations [101,102].

Recent works have established RIS as an effective secrecy tool in a variety of practical settings. The authors in [103] analyzed RIS-aided links with mobile or unmanned aerial vehicle (UAV) eavesdroppers and show that RIS phase control can substantially increase ergodic secrecy capacity by dispersing Eve’s channel gains and reducing its coherent combining opportunities. UAV-mounted RIS and jointly optimize UAV trajectory and RIS phase profiles have been studied by [104] to maximize secrecy rate under realistic CSI uncertainty, demonstrating that mobility plus programmable reflections yields large secrecy improvements over static deployments. Advancing physical-layer security in RIS-assisted wireless systems, the authors in [101] address multi-user secrecy in intelligent reflective surfaces (IRS)-aided systems and propose joint beamformer–phase optimization algorithms that balance secrecy across users while respecting RIS hardware constraints. These works make two points clear for lattice-based secrecy: (i) RIS can amplify the channel asymmetry that lattice coset coding exploits, and (ii) RIS constraints (finite phase resolution, training overhead, and imperfect CSI) must be explicitly modeled when designing algebraic codes for RIS channels.

From the lattice-coding side, there is a compact set of rigorous results that translate naturally to RIS contexts. A lattice design criterion presented in [105] for wiretap channels correlates algebraic diversity and minimum distance with secrecy performance. This criterion pinpoints algebraic parameters that increase the eavesdropper’s flatness factor under conditions where the effective channel is favorable to the legitimate receiver. In [102], Near-Field RIS secure lattice constructions were developed for compound channels, with the investigation revealing that control over the flatness factor facilitates information-theoretic secrecy, even amidst channel uncertainty. Also, ref. [106] constructs almost universal modular coding structures that achieve secrecy capacity and enable quantum-resistant authentication and key agreement, making them compatible with lattice-based RIS-assisted 6G networks. It considers a range of fading wiretap channels, demonstrating robustness of algebraic lattices to channel variations. Although these lattice works were not written specifically for RIS, their secrecy metrics are channel-geometric: an RIS that reshapes singular vectors and path gains directly changes the lattice embedding seen by the receiver and Eve. Therefore, the algebraic design aspects identified in these works (index, discriminant, unit rotations, and modularity) can be jointly optimized with the RIS phase shifts.

Putting these strands together suggests concrete design recipes and research directions:

- Co-optimization of RIS phases and lattice rotations. Treat RIS phase settings and lattice unit rotations as coupled variables in a secrecy objective that directly includes the flatness factor or secrecy gain. The RIS can be used to align the receiver’s effective channel with lattice directions that maximize minimum distance, while simultaneously dispersing Eve’s projections to increase the flatness factors.
- Robust coset scheduling under quantized RIS control. Practical RIS hardware has finite phase resolution and limited update rates. Design cyclotomic p-modular coset families whose decoding regions are tolerant to small phase errors, and schedule coset randomization across coherence blocks so that Eve cannot average out RIS-induced randomness.
- Pilot and training design using lattice structure. Pilot contamination and insecure feedback are major RIS vulnerabilities. Lattice-structured pilots (pilots drawn from carefully chosen coset representatives) can make spoofing harder and enable joint pilot–phase estimation algorithms that exploit algebraic redundancy to detect active attacks.
- Mobility and time-varying RIS strategies. For UAV-RIS or mobile RIS platforms, synchronize coset changes with RIS trajectory/phase updates so that Eve’s channel observations are decorrelated over time; this temporal diversity compounds the spatial confusion provided by lattice cosets.
- Complexity-aware implementations. High-dimension lattices yield strong secrecy gains but heavy decoding cost. Use block-diagonal or layered lattice constructions that match RIS-created subspaces, enabling per-subspace decoding with moderate complexity while preserving global secrecy metrics.

There are also important practical challenges. Accurate CSI is essential for effective RIS phase optimization; imperfect CSI reduces the ability to align lattice directions and can leak structure to Eve if not handled carefully [107]. Channel estimation overhead for RIS-assisted links is nontrivial, and secure feedback channels for RIS control are required to prevent adversarial reconfiguration. Finally, hardware impairments (phase noise, element coupling, and quantization) change the effective lattice seen by receivers; algebraic constructions with built-in robustness (e.g., modular lattices with favorable discriminants and unit groups) are promising candidates to absorb such distortions [105,108].

In short, RIS and lattice codes are complementary: RIS provides a programmable, geometric degree of freedom that can be exploited to shape the lattice channel in favor of secrecy, while algebraic lattice constructions provide provable, information-theoretic secrecy metrics that guide RIS optimization. Because the studies that jointly analyze lattice codes and RIS secrecy are still sparse, a high-impact research agenda is to develop rigorous flatness-factor and secrecy-gain analyses for RIS-transformed lattice channels, accompanied by practical co-design algorithms that respect RIS quantization, training, and complexity constraints.

### 7.3. Potential Machine-Learning Integration

Machine learning is increasingly leveraged in wireless systems, and physical-layer security is no exception. Deep learning can, for example, learn to optimize transmit strategies or to detect eavesdropping anomalies in complex environments. In the context of lattice-based PLS, ML can aid both encoding and decoding. On the encoding side, reinforcement learning has been proposed to tune IRS or UAV parameters for secrecy, effectively learning good RIS configurations without full CSI. For instance, ref. [109] apply a deep deterministic policy gradient (DDPG) algorithm to jointly optimize the phases of UAV-mounted RIS and achieve a high secrecy rate in a complex cell-free MIMO scenario. This demonstrates that ML can help solve the non-convex optimization problems arising in coded secure transmission. On the decoding side, one might employ neural-network-based lattice decoders that approximate maximum-likelihood decoding for high-dimensional lattices, reducing complexity. Preliminary work on deep decoders for lattice codes suggests that neural nets can learn to invert lattice quantization under noise [110,111].

Moreover, ML can assist in estimating the eavesdropper’s channel or in implementing privacy amplification: for example, a neural network could predict the secrecy outage probability of a given lattice code in fading environments, and adapt the code parameters (such as choosing among different cyclotomic lattices); accordingly, [112] presents a dynamic range query privacy-preserving scheme for blockchain-enhanced smart grid based on lattice. Conversely, one must be cautious of adversarial machine learning: an intelligent eavesdropper might use ML to infer the lattice structure or to mount new attacks.

The literature on learning for secure communications is rapidly growing. In addition to the DDPG [113], there are broad surveys of ML for wireless security. For example, ref. [114] overview 5G-and-beyond privacy-preserving data-driven learning models for emerging communication networks and mention the use of learning algorithms for joint communication and physical-layer security. The authors in [115] study intelligent decentralized federated graph learning with lightweight zero trust architecture for next-generation networking security, making it compatible with lattice-based architectures. These and related works indicate that ML can augment lattice-based schemes, but also that integrating ML introduces new trade-offs: learning models need data and training time, and their decisions may lack provable guarantees (in contrast to information-theoretic lattice designs). Ensuring the reliability and interpretability of ML-aided PLS algorithms is thus an open challenge.

### 7.4. Open Problems

The integration of modular lattice codes into MIMO, RIS, and ML-based systems raises many research questions. First, designing optimal lattice codes for MIMO wiretap channels remains unsolved: how to choose *p*-modular lattices that maximize secrecy gain under multi-antenna constraints and imperfect CSI? Closed-form design criteria (generalizing flatness factor) for fading MIMO channels are still lacking. Second, implementation complexity is a major issue: effective high-dimensional lattice decoding in real time (especially over MIMO) is challenging. Hybrid schemes that combine lattices with more conventional MIMO precoding need exploration. Third, in RIS-assisted systems, joint optimization of lattice cosets and RIS phases is a new design space. How to quantize phase shifts to preserve lattice secrecy, and how to coordinate distributed RIS elements for cooperative secrecy coding, are open problems. Additionally, the discrete nature of p-modular lattices (integer ring structure) may need adaptation to the analog domain of RIS (like continuous phase).

From a machine learning perspective, integrating data-driven methods with algebraic coding raises questions of generalization and security: can learning algorithms reliably optimize lattice-coded transmissions for secrecy across diverse channel conditions? How can we guard against learned models that may overfit or be fooled by adversaries? The intersection of ML and lattice coding is largely unexplored. Finally, multi-user and network scenarios (multiple eavesdroppers, relays, or feedback links) have not been studied with cyclotomic lattice codes. Extending these codes to distributed networks (for example, using compute-and-forward or cooperative jamming with lattices) is a rich avenue for future work. Modern intelligent wireless platforms offer new tools for physical-layer security, but they also introduce new vulnerabilities and design complexities. The algebraic and geometric features of p≡1(mod4) cyclotomic lattices (e.g., rich unit groups, well-roundedness) may be leveraged to meet these challenges. For instance, their flatness factors could potentially be engineered to remain low over the enhanced channels in RIS environments, or ML could be used to approximate optimal decoding. Nevertheless, rigorous analysis and prototypes are needed to validate these ideas in practice.

## 8. Conclusions

This article has provided a comprehensive survey bridging the theoretical richness of algebraic lattice theory with its critical applications in physical-layer security for wireless communications. We established the foundational models of wireless wiretap channels and detailed essential information-theoretic secrecy metrics such as secrecy gain and flatness factor, which remain central to evaluating the confidentiality of lattice-coded transmissions. Our exposition moved from algebraic number theory fundamentals and Construction A to modular and unimodular lattice families, consolidating classical and modern results into a unified framework that highlights the deep interplay between algebraic structures and secure communication. In addition, we reviewed a newly proposed family of *p*-modular lattices constructed from cyclotomic number fields $Q(\zeta_{p})$ for primes p≡1(mod4), developed through a generalized Construction A methodology. We rigorously characterized their algebraic and geometric properties and established a non-existence theorem for *p*-modular lattices arising from prime-power cyclotomic fields $Q(\zeta_{p^{n}})$ with n>1. These results provide concrete engineering design principles, identifying both viable construction pathways and fundamental structural limitations for future lattice code development.

Another future research direction is the integration of recent advances in the theory of indefinite theta series and modular completions. Since these *p*-modular constructions naturally yield lattices with mixed signature, the classical theta series fails to converge on the unit circle, rendering traditional secrecy metrics inapplicable. By drawing on the modern framework of Vignéras’ differential equation and the generalized error functions $E_{1},M_{1},E_{2},M_{2}$, we highlighted how modularly completed indefinite theta series offer a principled analytic replacement. This perspective opens the door to defining secrecy-relevant quantities for indefinite lattices and suggests new directions for characterizing flatness, smoothing, and eavesdropper performance through the geometry of time-like and space-like directions.

The broader implications of this research are significant for next-generation wireless systems. The algebraic structure, modular behavior, and potential secrecy advantages of these lattices make them promising candidates for integration into advanced communication paradigms such as Massive MIMO, RIS-aided architectures, and machine-learning-driven optimization. By combining algebraic number theory, modular analysis, and physical-layer security, this paper serves as both a tutorial and a design resource, guiding researchers and engineers in leveraging sophisticated mathematical tools to build robust, analyzable, and secure wireless networks for the future.

## Figures and Tables

**Table 1:** Theta series of some exceptional lattices.

Lattice Λ	Theta Series $\Theta_{\Lambda }$
Cubic lattice $Z^{n}$	$\vartheta_{3}^{n}$
Checkerboard lattice $D_{n}$	$\frac{1}{2}\left(\vartheta_{3}^{n}+\vartheta_{4}^{n}\right)$
Gosset lattice $E_{8}$	$\frac{1}{2}\left(\vartheta_{2}^{8}+\vartheta_{3}^{8}+\vartheta_{4}^{8}\right)$
Leech lattice $\Lambda_{24}$	$\frac{1}{8}{\left(\vartheta_{2}^{8}+\vartheta_{3}^{8}+\vartheta_{4}^{8}\right)}^{3}-\frac{45}{16}{\left(\vartheta_{2}\cdot \vartheta_{3}\cdot \vartheta_{4}\right)}^{8}$

**Table 2:** Secrecy-relevant indicators for classical and proposed lattice families, including signature and definiteness.

Lattice Family	Construction Setting	Field Signature	Is Definite?	Secrecy Implications
Unimodular lattices	Integral, positive-definite	(n,0)	Yes	Classical secrecy gain and flatness factor available; strong shaping
*q*-modular lattices	Integral, positive-definite	(n,0)	Yes	Classical secrecy metrics applicable; good confusion at Eve
Lattices from cyclotomic fields	Canonical embedding or Construction A	CM: $\left(0,\frac{n}{2}\right)$ or real: (n,0)	Yes	Classical secrecy metrics applicable (positive-definite theta series)
Proposed *p*-modular lattices	As in Theorem 13: $K=Q(\zeta_{p})$, $\alpha =1/\sqrt{p}$	CM: $\left(0,\frac{p-1}{2}\right)$	No, mixed signature $\left(n_{+},n_{-}\right)$	Classical secrecy gain fails for analytic model; modularly completed indefinite theta series required for secrecy-relevant quantities
Prime-power cyclotomic fields $Q(\zeta_{p^{r}})$, r>1	No modular Construction A lattice	CM: $\left(0,\frac{\phi (p^{r})}{2}\right)$	Not applicable	Not suitable for PLS under generalized Construction A

## Data Availability

No new data were created or analyzed in this study.

## Abbreviations

The following abbreviations are used in this manuscript:

AN	Artificial Noise
APs	Access Points
AWGN	Additive White Gaussian Noise
CSI	Channel State Information
DDPG	Deep Deterministic Policy Gradient
IRS	Intelligent Reflecting Surfaces
LDA	Integer Low-Density Lattices
LDLC	Low-Density Lattice Codes
MIMO	Multiple-Input Multiple-Output
ML	Machine Learning
PLS	Physical-Layer Security
QAM	Quadrature Amplitude Modulation
RIS	Reconfigurable Intelligent Surfaces
RSMA	Rate-Splitting Multiple Access
SNR	Signal to Noise Ratio
UAV	Unmanned Aerial Vehicle
uRLLC	Ultra-Reliable Low-Latency Communications
VNR	Volume-to-Noise Ratio

## Appendix A. Proofs

### Appendix A.1. Theorem 13

**Proof** **of Theorem 13.** Since $G=\{\sigma_{1},\ldots ,\sigma_{p-1}\}$, with $\sigma_{i}:\zeta_{p}\mapsto \zeta_{p}^{i}$, is the Galois group of K/Q, *K* is a totally imaginary number field and it is a quadratic extension of $K^{+}=Q\left(\zeta_{p}+\zeta_{p}^{-1}\right)$, which is a totally real number field. Thus, *K* is a CM field. The minimal polynomial of $\zeta_{p}$ is $\Phi_{p}\left(X\right)=1+X+X^{2}+\ldots +X^{p-1}$ and $p|\Phi_{p}\left(1\right)$. This indicates $\left(X-1\right)|{\bar{\Phi }}_{p}\left(X\right)$, where ${\bar{\Phi }}_{p}=\Phi_{p}\;\left(\mathrm{mod}\;p\right)$. Using Proposition 2, $P=pO_{K}+\left(1-\zeta_{p}\right)O_{K}$ is a prime ideal of $O_{K}$ and $O_{K}/P≅F_{p}$. It can be proved that P is totally ramified and $pO_{K}=P^{p-1}$ [47], Lemma 5.3, p. 123. We also conclude from the Kronecker–Weber theorem that $\sqrt{p}\in K$ and consequently $\alpha =\frac{1}{\sqrt{p}}$ is in *K*. Thus, $b_{\alpha }$, with $b_{\alpha }\left(x,y\right)=\sum_{i=1}^{N}{\mathrm{Tr}}_{K/Q}\left(\alpha x_{i}{\bar{y}}_{i}\right)$ for $x,y\in O_{K}^{N}$, is a symmetric bilinear form. The dual of $\rho^{-1}\left(C\right)$ is given by $\left(\rho^{-1}{\left(C\right)}^{*},b_{\alpha }\right)$, where $\rho^{-1}{\left(C\right)}^{*}=\left\{x\in K^{N}\;:\;b_{\alpha }\left(x,y\right)\in Z,\;\;\mathrm{for}\;\;\mathrm{all}\;\;y\in \rho^{-1}\left(C\right)\right\}$. First, we prove that $\frac{1}{\sqrt{p}}\rho^{-1}\left(C\right)=\rho^{-1}{\left(C\right)}^{*}$ as Z-modules. Take any $x\in \frac{1}{\sqrt{p}}\rho^{-1}\left(C\right)=\frac{1}{\sqrt{p}}\rho^{-1}\left(C^{⊥}\right)$, $x=\frac{1}{\sqrt{p}}x^{\prime }$ with $x^{\prime }\in \rho^{-1}\left(C^{⊥}\right)$, and $y\in \rho^{-1}\left(C\right)$, then

$$\begin{array}{ccc} \rho (x^{\prime }\cdot y) & = & \rho \left(\sum_{i=1}^{N}x_{i}^{\prime }y_{i}\right)=\sum_{i=1}^{N}\rho \left(x_{i}^{\prime }\right)\rho \left(y_{i}\right)\\ & = & \rho \left(x^{\prime }\right)\cdot \rho \left(y\right)=0\in F_{p}, \end{array}$$

which gives $x^{\prime }\cdot y\equiv 0\;\left(\mathrm{mod}\;P\right)$. As P is totally ramified, by the proof from [49], $\bar{\gamma }=\gamma \;\left(\mathrm{mod}\;P\right)$, for all $\gamma \in O_{K}$. Indeed, using $O_{K}/P≅F_{p}$, we can write $\gamma =\gamma^{\prime }+\gamma^{\prime \prime }$, with $\gamma^{\prime }\in \left\{0,1,\ldots ,p-1\right\}$ and $\gamma^{\prime \prime }\in P$ and since P is the only prime above *p*, $\bar{P}=P$ and we have $\bar{\gamma^{\prime \prime }}\in P$. Thus

$$\bar{\gamma }={\bar{\gamma }}^{\prime }+{\bar{\gamma }}^{\prime \prime }=\gamma^{\prime }+{\bar{\gamma }}^{\prime \prime }\equiv \gamma^{\prime }\equiv \gamma \;\;\left(\;\mathrm{mod}\;\;\;P\right).$$

Then, we can conclude

$$x^{\prime }\cdot \bar{y}\equiv x^{\prime }\cdot y\;\;\left(\mathrm{mod}\;\;\;P\right)⟹x^{\prime }\cdot \bar{y}\in P.$$

As P is the only prime above *p*, we have $\sigma_{i}\left(x^{\prime }\cdot \bar{y}\right)\in P$, for i=1,…,p−1. Hence, ${\mathrm{Tr}}_{K/Q}\left(x^{\prime }\cdot \bar{y}\right)\in P\cap Z=pZ$ (see Remark 2) and

$$\begin{array}{ccc} b_{\alpha }\left(x,y\right) & = & \sum_{i=1}^{N}{\mathrm{Tr}}_{K/Q}\left(\alpha x_{i}{\bar{y}}_{i}\right)={\mathrm{Tr}}_{K/Q}\left(\alpha x\cdot \bar{y}\right)={\mathrm{Tr}}_{K/Q}\left(\frac{\alpha }{\sqrt{p}}x^{\prime }\cdot \bar{y}\right)\\ & = & \frac{1}{p}{\mathrm{Tr}}_{K/Q}\left(x^{\prime }\cdot \bar{y}\right)\in \frac{1}{p}pZ. \end{array}$$

Hence, $b_{\alpha }\left(x,y\right)\in Z$ and we have proved that $\frac{1}{\sqrt{p}}\rho^{-1}\left(C\right)\subseteq \rho^{-1}{\left(C\right)}^{*}$. On the other hand, $\mathrm{vol}\left(\rho^{-1}\left(C\right)\right)={\left|d_{K}\right|}^{N/2}p^{N-k}N{\left(\alpha \right)}^{N/2}$, with $k=\frac{N}{2}$, $|d_{K}|=p^{p-2}$ and $N\left(\alpha \right)=\frac{1}{p^{\frac{p-1}{2}}}$ that is obtained from $\frac{1}{p^{p-1}}=N\left(\frac{1}{p}\right)=N\left(\alpha^{2}\right)={\left(N\left(\alpha \right)\right)}^{2}$, implies

(A1) $$\begin{array}{ccc} \mathrm{vol}\left(\frac{1}{\sqrt{p}}\rho^{-1}\left(C\right)\right) & = & \mathrm{vol}\left(\rho^{-1}\left(C\right)\right)\left|\frac{\rho^{-1}\left(C\right)}{\frac{1}{\sqrt{p}}\rho^{-1}\left(C\right)}\right|=p^{\frac{(p-1)N}{4}}{\left(\frac{1}{\sqrt{p}}\right)}^{(p-1)N} \end{array}$$

(A2) $$\begin{array}{ccc} & = & p^{-\frac{(p-1)N}{4}}, \end{array}$$

and

$$\mathrm{vol}\left(\rho^{-1}{\left(C\right)}^{*}\right)=\frac{1}{\mathrm{vol}(\rho^{-1}\left(C\right))}=p^{-\frac{(p-1)N}{4}}.$$

Thus, we have $\rho^{-1}{\left(C\right)}^{*}=\frac{1}{\sqrt{p}}\rho^{-1}\left(C\right)$. Define

$$\begin{array}{ccc} h:(\rho^{-1}\left(C\right),b_{\alpha }) & \to & \left(\rho^{-1}{\left(C\right)}^{*},b_{\alpha }\right)\\ x & \mapsto & \frac{1}{\sqrt{p}}x. \end{array}$$

Due to the above discussion, *h* is a Z-linear bijection. Let $x,y\in \rho^{-1}\left(C\right)$,

$$\begin{array}{ccc} p\cdot b_{\alpha }\left(h\left(x\right),h\left(y\right)\right) & = & p\cdot {\mathrm{Tr}}_{K/Q}\left(\sum_{i=1}^{N}\alpha h{\left(x\right)}_{i}{\bar{h(y)}}_{i}\right)\\ & = & p\cdot {\mathrm{Tr}}_{K/Q}\left(\sum_{i=1}^{N}\alpha \frac{x_{i}}{\sqrt{p}}\frac{\bar{y_{i}}}{\sqrt{p}}\right)\\ & = & {\mathrm{Tr}}_{K/Q}\left(\sum_{i=1}^{N}\alpha x_{i}\bar{y_{i}}\right)=b_{\alpha }\left(x,y\right). \end{array}$$

This completes the proof. □

### Appendix A.2. Proposition 10

**Proof** **of Proposition 10.** Using Proposition 5, a generator matrix for $\left(\rho^{-1}\left(C\right),b_{\alpha }\right)$, with $\alpha =1/\sqrt{5}$, is

(A3) $$M_{C}=\left[\begin{array}{cc} M & A⊗M\\ 0_{4\times 4} & M_{p} \end{array}\right]\left(I_{2}⊗D_{\alpha }\right),$$

where $M,M_{p}$ and A are real matrices and the matrix $D_{\alpha }$ is the diagonal matrix $\mathrm{diag}\left(\sqrt{\sigma_{1}\left(\alpha \right)},\sqrt{\sigma_{2}\left(\alpha \right)},\sqrt{\sigma_{3}\left(\alpha \right)},\sqrt{\sigma_{4}\left(\alpha \right)}\right)$. Thus, it is enough to compute $\sigma_{i}\left(\alpha \right)$, for i=1,…,4. Using quadratic Gauss sums in (62) and $\zeta_{5}^{4}=-1-\zeta_{5}-\zeta_{5}^{2}-\zeta_{5}^{3}$, we can write $\alpha =\sqrt{5}/5$ as follows:

(A4) $$\frac{\sqrt{5}}{5}=\frac{\zeta_{5}-\zeta_{5}^{2}-\zeta_{5}^{3}+\zeta_{5}^{4}}{5}=\frac{-1-2\zeta_{5}^{2}-2\zeta_{5}^{3}}{5}.$$

Consequently, we have

$$\begin{array}{ccc} \sigma_{1}\left(\alpha \right) & = & \sigma_{2}\left(\alpha \right)=\frac{-1-2\zeta_{5}^{2}-2\zeta_{5}^{3}}{5}=\frac{\sqrt{5}}{5}=\alpha ,\\ \sigma_{3}\left(\alpha \right) & = & \sigma_{4}\left(\alpha \right)=\frac{-1-2\zeta_{5}^{4}-2\zeta_{5}}{5}=\frac{1+2\zeta_{5}^{2}+2\zeta_{5}^{3}}{5}=-\alpha . \end{array}$$

Using the above equations and (57), we can check that $N\left(\alpha \right)=5^{-2}$ and ${\mathrm{Tr}}_{K/Q}\left(\alpha \right)=0$. Due to the definition of ρ in Remark 2, we have $\sqrt{5}\in P$, because $\rho \left(\sqrt{5}\right)=-1-2-2\;\left(\mathrm{mod}\;5\right)=0$. We have

$$D_{\alpha }=\mathrm{diag}\left(\sqrt{\alpha },\sqrt{\alpha },\sqrt{-\alpha },\sqrt{-\alpha }\right)=\mathrm{diag}\left(\sqrt{\alpha },\sqrt{\alpha },i\sqrt{\alpha },i\sqrt{\alpha }\right).$$

This proves the result. □

### Appendix A.3. Theorem 14

**Proof** **of Theorem 14.** If r=1 and d=1, then $K=Q(\zeta_{p})$ and the result follows from Proposition 8. Now assume that $\left(\rho^{-1}\left(C\right),b_{1/p}\right)$ is a *d*-modular lattice. Then, due to the definition of modular lattices, we have $\frac{1}{\sqrt{d}}\rho^{-1}\left(C\right)={\left(\rho^{-1}\left(C\right)\right)}^{*}$. Consequently, $\mathrm{vol}\left(\frac{1}{\sqrt{d}}\rho^{-1}\left(C\right)\right)=\mathrm{vol}\left({\left(\rho^{-1}\left(C\right)\right)}^{*}\right)$. We have $\mathrm{vol}\left({\left(\rho^{-1}\left(C\right)\right)}^{*}\right)=1/\mathrm{vol}\left(\rho^{-1}\left(C\right)\right)$ that implies $d^{\frac{-nN}{2}}{\left(\mathrm{vol}\left(\rho^{-1}\left(C\right)\right)\right)}^{2}=1$. It is enough to compute $\mathrm{vol}(\rho^{-1}\left(C\right))$. To this end, we have $\mathrm{vol}\left(\rho^{-1}\left(C\right)\right)=\sqrt{\left|\mathrm{disc}(\rho^{-1}\left(C\right))\right|}=\sqrt{\Delta^{N}p^{2N-2k-nN}}$, where $\Delta =|d_{K}|=p^{p^{r-1}\left(pr-r-1\right)}$. Thus, we have

(A5) $$\begin{array}{ccc} d^{\frac{-nN}{2}}p^{2N-2k-nN}p^{Np^{r-1}\left(pr-r-1\right)} & = & 1. \end{array}$$

We conclude that *d* is 1 or *p*. Let d=p and apply $n=p^{r-1}\left(p-1\right)$ in (A5). We have

$$\begin{array}{ccc} \frac{-p^{r-1}\left(p-1\right)N}{2}+2N-2k-p^{r-1}\left(p-1\right)N+Np^{r-1}\left(pr-r-1\right) & = & 0. \end{array}$$

Simplifying the above equation gives the following relation

$$\begin{array}{ccc} N\left[3\left(1-p\right)p^{r-1}+2p^{r-1}\left(pr-r-1\right)+4\right] & = & 4k. \end{array}$$

Since C is a self-dual code, k=N/2 and we have

$$\begin{array}{ccc} 2 & = & 3\left(1-p\right)p^{r-1}+2p^{r-1}\left(pr-r-1\right)+4\\ & = & p^{r-1}\left(1-3p+2pr-2r\right)+4. \end{array}$$

Finally, we have $p^{r-1}\left(1-3p+2pr-2r\right)=-2$ which implies r=2 and p=2 or r=1 and p=1 and both of these cases are contradictions. Thus, d=1 and it is enough to show that r=1. In this case, $\rho^{-1}\left(C\right)$ is unimodular and we have $\rho^{-1}\left(C^{⊥}\right)={\left(\rho^{-1}\left(C\right)\right)}^{*}$ and consequently $\mathrm{vol}\left(\rho^{-1}\left(C^{⊥}\right)\right)=\mathrm{vol}\left({\left(\rho^{-1}\left(C\right)\right)}^{*}\right)$, which implies

(A6) $$\begin{array}{ccc} k-\frac{N}{2}\left(p^{r-1}\left(rp-r-p\right)+2\right) & = & k+\frac{N}{2}\left(p^{r-1}\left(rp-r-p\right)\right). \end{array}$$

We conclude that $p^{r-1}\left(rp-r-p\right)=-1$ which is possible if and only if r=1. □

### Appendix A.4. Theorem 15

**Proof** **of Theorem 15.** If r=1 and d=1, then $K^{+}=Q\left(\zeta_{p}+\zeta_{p}^{-1}\right)$ and the result follows from Proposition 9. Now, assume that $\left(\rho^{-1}\left(C\right),b_{1/p}\right)$ is a *d*-modular lattice. Then, due to the definition of modular lattices, we have $\frac{1}{\sqrt{d}}\rho^{-1}\left(C\right)={\left(\rho^{-1}\left(C\right)\right)}^{*}$. Consequently, $\mathrm{vol}\left(\frac{1}{\sqrt{d}}\rho^{-1}\left(C\right)\right)=\mathrm{vol}\left({\left(\rho^{-1}\left(C\right)\right)}^{*}\right)$. We have $\mathrm{vol}\left({\left(\rho^{-1}\left(C\right)\right)}^{*}\right)=1/\mathrm{vol}\left(\rho^{-1}\left(C\right)\right)$ that implies $d^{\frac{-nN}{2}}{\left(\mathrm{vol}\left(\rho^{-1}\left(C\right)\right)\right)}^{2}=1$. It is enough to compute $\mathrm{vol}(\rho^{-1}\left(C\right))$. Similarly as before, we have $\mathrm{vol}\left(\rho^{-1}\left(C\right)\right)=\sqrt{\left|\mathrm{disc}(\rho^{-1}\left(C\right))\right|}=\sqrt{\Delta^{N}p^{2N-2k-nN}}$, where $\Delta =|d_{K^{+}}|=p^{\frac{p-1}{2}\left(\left(r+1\right)p^{r-1}-\frac{p^{r}-1}{p-1}\right)-1}$. Thus, we have

(A7) $$\begin{array}{ccc} d^{\frac{-nN}{2}}p^{2N-2k-nN}p^{N\left[\frac{p-1}{2}\left(\left(r+1\right)p^{r-1}-\frac{p^{r}-1}{p-1}\right)-1\right]} & = & 1. \end{array}$$

We conclude that *d* is 1 or *p*. Let d=p, and apply $n=\frac{p^{r-1}\left(p-1\right)}{2}$ in (A7). We have

$$\begin{array}{c} \frac{p^{r-1}\left(1-p\right)N}{4}+N+\frac{p^{r-1}\left(1-p\right)N+N\left(p-1\right)\left(\left(r+1\right)p^{r-1}-\frac{p^{r}-1}{p-1}\right)}{2}=2k. \end{array}$$

Since C is a self-dual code, k=N/2 and we have

$$\begin{array}{ccc} 0 & = & \frac{N(p-1)}{4}\left[-3p^{r-1}+2\left(r+1\right)p^{r-1}-\frac{2(p^{r}-1)}{p-1}\right]\\ & = & \frac{N}{4}\left[-3\left(p-1\right)p^{r-1}+2\left(r+1\right)\left(p-1\right)p^{r-1}-2\left(p^{r}-1\right)\right]\\ & = & \frac{N}{4}\left[p^{r}\left(2r-3\right)+p^{r-1}\left(1-2r\right)+2\right]\\ & = & \frac{N}{4}\left[p^{r-1}\left(2pr-3p+1-2r\right)+2\right]. \end{array}$$

Thus, we have $p^{r-1}\left(1-3p+2pr-2r\right)=-2$ which implies r=2 and p=2 or r=1 and p=1 and both of these cases are contradictions. Thus, d=1 and it is enough to show that r=1. In this case, $\rho^{-1}\left(C\right)$ is unimodular and we have $\rho^{-1}\left(C^{⊥}\right)={\left(\rho^{-1}\left(C\right)\right)}^{*}$ and consequently $\mathrm{vol}\left(\rho^{-1}\left(C^{⊥}\right)\right)=\mathrm{vol}\left({\left(\rho^{-1}\left(C\right)\right)}^{*}\right)$, which implies

(A8) $$\begin{array}{ccc} k+\frac{N\left[p^{r-1}\left(rp-r-p\right)-1\right]}{4} & = & k-\frac{N\left[p^{r-1}\left(rp-r-p\right)+3\right]}{4}. \end{array}$$

We conclude that $p^{r-1}\left(rp-r-p\right)=-1$ which is possible if and only if r=1. □
